# Effective hybrid feature selection using different bootstrap enhances cancers classification performance

**DOI:** 10.1186/s13040-022-00304-y

**Published:** 2022-09-30

**Authors:** Noura Mohammed Abdelwahed, Gh. S. El-Tawel, M. A. Makhlouf

**Affiliations:** 1 Department of Information Systems, Faculty of Computers and Informatics, Suez Canal University, Ismailia, Egypt; 2 Department of Computer Science, Faculty of Computers and Informatics, Suez Canal University, Ismailia, Egypt

**Keywords:** Machine Learning, Random Forest feature importance, Recursive feature elimination and its disadvantages, Over-fitting, Learning algorithms, High dimensional data

## Abstract

**Background:**

Machine learning can be used to predict the different onset of human cancers. Highly dimensional data have enormous, complicated problems. One of these is an excessive number of genes plus over-fitting, fitting time, and classification accuracy. Recursive Feature Elimination (RFE) is a wrapper method for selecting the best subset of features that cause the best accuracy. Despite the high performance of RFE, time computation and over-fitting are two disadvantages of this algorithm. Random forest for selection (RFS) proves its effectiveness in selecting the effective features and improving the over-fitting problem.

**Method:**

This paper proposed a method, namely, positions first bootstrap step (PFBS) random forest selection recursive feature elimination (RFS-RFE) and its abbreviation is PFBS- RFS-RFE to enhance cancer classification performance. It used a bootstrap with many positions included in the outer first bootstrap step (OFBS), inner first bootstrap step (IFBS), and outer/ inner first bootstrap step (O/IFBS). In the first position, OFBS is applied as a resampling method (bootstrap) with replacement before selection step. The RFS is applied with bootstrap = false i.e., the whole datasets are used to build each tree. The importance features are hybrid with RFE to select the most relevant subset of features. In the second position, IFBS is applied as a resampling method (bootstrap) with replacement during applied RFS. The importance features are hybrid with RFE. In the third position, O/IFBS is applied as a hybrid of first and second positions. RFE used logistic regression (LR) as an estimator. The proposed methods are incorporated with four classifiers to solve the feature selection problems and modify the performance of RFE, in which five datasets with different size are used to assess the performance of the PFBS-RFS-RFE.

**Results:**

The results showed that the O/IFBS-RFS-RFE achieved the best performance compared with previous work and enhanced the accuracy, variance and ROC area for RNA gene and dermatology erythemato-squamous diseases datasets to become 99.994%, 0.0000004, 1.000 and 100.000%, 0.0 and 1.000, respectively.

**Conclusion:**

High dimensional datasets and RFE algorithm face many troubles in cancers classification performance. PFBS-RFS-RFE is proposed to fix these troubles with different positions. The importance features which extracted from RFS are used with RFE to obtain the effective features.

## Introduction

Artificial intelligence (AI) is a science that plays an important role in all fields, especially in the biomedical field, and it aims to simulate reality [1, 2]. Different AI applications have been applied in this field for 20 years due to many factors, including the availability of different datasets in this field, computer devices with high capabilities and arithmetic algorithms [2]. AI has great importance, as a survey has proven that it has great effectiveness in health, and it will outperform the performance of specialists in this field. In addition, it has proven effective in cancer research [2]. Furthermore, AI has become providing human specialists with many information and accordingly, the decision is taken, as it has become one of the most important elements in the medical team [2]. It also works to improve accuracy, speed up diagnosis and discover features or genes affecting cancer as recommendations for human specialists to take into consideration [2]. AI is considered a second decision that helps the specialist make their decision [2]. AI differs from the manual method because it provides human specialists with more information and details. Its diagnosis is more accurate and efficient and does not require more labor.

The manual method may be stressful for the patient, as it puts him under great pressure and takes more time to know the results of the sample, which makes him tense [3]. Cancer has become very widespread in recent times, as it has become a major cause of disease and death [4]. It can be defined as a group of more than one disease due to abnormal cell growth or changes in genes, and it can occur anywhere in the body [5]. Many factors cause cancer including [6]: - (1) tobacco consumption, (2) poor diet, (3) lack of physical activity, (4) alcohol, (5) radiation, (6) infection, (7) genetic factors, (8) smoking and (9) age [6]. There are many different types of human cancer, but in this paper, we used some types that included Breast Invasive Carcinoma (BR), Bladder urothelial carcinoma (BL), Colon and rectum (CO), Glioblastoma multiform (GB), Head and neck squamous cell (HN), Kidney renal clear-cell (KI), Parkinson’s disease (PD), Prostate adenocarcinoma (PRAD) and Lung adenocarcinoma (LUAD).

There are enormous problems in big datasets involved in the features numbers, fitting time, classification accuracy, and model performance. Feature selection is a process for selecting the most relevant features and discarding insignificant ones. Feature selection plays a vital role in many directions to enhance the model performance [7–9]. This process aims to select the most relevant subset r features from the original R features set (r < R) in given datasets [9]. R includes all features in a dataset. It suffers from many problems included in high dimension, noisy, repetitive and over-fitting. The ineffective features are deleted. These features diminish the classification accuracy and waste time. By deleting irrelevant features, all previous problems are solved and improved. Feature selection procedures have three major types: filter, wrapper [9, 10], and embedded [11]. Filter procedure selects the features by evaluating their relevance of features. These features are ranked in decreased order, and low-ranking features are omitted to obtain the most relevant features [12]. The filter approach can use many measures included in gain ratio, mutual information based feature selection (MIFS), information gain based feature selection (IGF), relaxed functional dependencies [9], and chi-square [10]. This procedure does not depend on any machine learning and is faster than the wrapper procedure. Despite its simplicity, it suffers from an over-fitting problem. The best subset of features is selected depending on machine learning to estimate this subset [9, 10]. This procedure suffers from expensive computationally when applied on high dimensions. On the other hand, it guarantees to select the most relevant and effective subset of features. Feature selection is an integral part of the classification model in the embedded procedure. It is embedded in the phase of learning [11]. This procedure has many advantages, including being less computationally expensive, reducing over-fitting problems, and selecting the most accurate features. In this direction, we adopted the integration of wrapper procedure with embedded one to select the relevant features using proposed methods to minimize the previous drawbacks and maximize the classification accuracy.

Selecting influencing features is an effective step in the classification process to obtain accurate results. Many datasets always suffer from high dimensions problems, which negatively affect the model performance’s accuracy. The feature selection step is considered one of the processes that positively impact solving many problems facing different datasets. In this direction, many authors applied different feature selection algorithms to minimize processing time, over-fitting, maximize classification accuracy and find the most relevant features, which still need more researches to improve. Therefore, there are numerous different methods for feature selection to fix the previous drawbacks included in the filter, wrapper, and embedded methods. The filter method is simple, and it selects the features based on their ranking according to a class. Still, it suffers from over-fitting problems in high dimensions datasets and disregards feature dependencies. Elsadek et al. [12] proposed a method using IGF to classify six human cancer types based on DNA copy number variation (CNV) dataset. The proposed method selected 16,381 features as the most relevant features. More than one learning algorithm is applied, such as logistic regression (LR), support vector machine (SVM), random forest (RF), J48, neural network, bagging and dagging. LR learning algorithm achieved the best classification accuracy of about 85% and ROC area 0.965. Rajit et al. [13] proposed selecting best and select percentile filter methods. The proposed method used a breast cancer dataset. There are more than one learning algorithms are used. LR classifier achieved a better result. Furthermore, many filter methods are proposed by Pinar Yildirim [14]. Different filter methods are applied in Cfs Subset eval, principal component analysis (PCA), consistency subset eval, IGF, One-R attribute eval, and relief attribute eval. The proposed method used the Hepatitis datasets and proved that the Consistency Subset, IGF, One-R Attribute Eval, and Relief Attribute Eval filter methods achieved better results. In addition, Alirezanejad et al. [15] proposed a filter method for gene selection using two heuristic methods. These methods, namely, Xvariance and mutual congestion. The Xvariance gave the best results with the standard datasets, while mutual congestion enhanced the accuracy of high-dimensional datasets. Kuswanto et al. [16] proposed a comparison method for feature selection using different filtering methods. Three filtering methods included in MIFS, correlation based feature selection (CFS) and fast correlation based feature selection (FCBF) are applied. The results of these methods are forwarded to K-nearest neighbors (KNN) classifer. The results showed that the FCBF selected a small number of features, while other methods performed well. Furthermore, Ghasemi et al. [17] proposed a method using IGF and gini index to select important features. These features are used to early predict of heart disease. This proposed method aimed to minimize the dimension and maximize the performance of the diagnosis of heart disease with less medical experiments. Mahmood [18] proposed a method to minimize a dimension for facial expression recognition dataset. Two feature selection methods are applied to obtain minimum number of features included in Chi-Square and Relief-F. These methods selected the first highest six features. Four different classifiers are applied to evaluate the performance. In addition, Spencer et al. [19] proposed a method to predict heart disease dataset. Four proposed methods are used for feature selection included in ReliefF, Chi-squared, symmetrical uncertainty and PCA. Different machine learning classifiers are applied to create models for comparison. The best prediction with less subset of features is selected using Chi-Square. Mohamed et al. [20] proposed a method to obtain the most important subset of feature rather than the whole dataset. Chi-square, IG and Bat algorithm are applied for feature selection. Many varieties of classifiers are used to evaluate the model performance. Vikas et al. [21] proposed a method to minimize processing time and maximize classification accuracy using lung cancer detection. To select the most relevant features, Chi-square algorithm is applied. Two different classifiers are used to evaluate the performance included in SVM and RF.

Many authors applied wrapper methods to solve the optimization problems and to get the most important subset features using different datasets. AH et al. [22] proposed an algorithm using the wrapper approach. The proposed algorithm enhanced the basic salp swarm algorithm (SSA) to improve reliability, convergence speed, and classification accuracy. The algorithm was enhanced by adding inertia weight to achieve better results. Hegazy et al. [9] used the hybrid wrapper method by applying chaotic maps to improve the performance of the salp swarm algorithm (SSA) and overcome its drawbacks. To control the exploitation/exploration rates, they used five chaotic maps. The proposed algorithm (CSSA) was applied on twenty-seven datasets and gave the best results. Although it gave the best results using twenty-seven datasets, it did not achieve good results using high-dimensional datasets. Sanaa et al. [8] proposed a wrapper method included in particle swarm optimization (PSO) and genetic algorithm (GA) to classify six human cancers types using DNA CNV dataset. The hybrid proposed method was applied to minimize the features and maximize the classification accuracy. It selected 2051 features from 16,381 features. The selected features achieved 84.6% classification accuracy. However, it suffered from many problems included in over-fitting, fitting time, relevant features, and classification accuracy. RFE is considered a wrapper method for feature selection. It suffers from time-consuming, especially when using big data. Li et al. [23] proposed fixing the support vector machine recursive feature elimination (SVMRFE) problem. They first proposed random value-based oversampling as a resampling method. The proposed variable step size (VSSRFE) to speed up the feature selection process. Another method is proposed called linear SVM (LLSVM). The two proposed methods are used together for feature selection. Jeon et al. [24] proposed a hybrid RFE method using benchmark datasets. This proposed method used SVM-RFE, random forest RFE (RF-RFE), and gradient boosting machines RFE (GBM-RFE) methods which combined the feature-importance-based RFE methods. There were two types of weighting functions used in the proposed methods. The first type sums the weight of three proposed RFE methods, and the second one reflects the classification accuracies and weights of features. Rani et al. [25] proposed a hybrid wrapper method by integrating GA and RFE algorithms. This method is compared with other feature selection methods. The proposed method improved the classification performance after canceling irrelevant features. Zvarevashe et al. [26] proposed a method to select the most relevant subset features using RFE algorithm based on RF. The proposed method was compared with a deep learning algorithm. It proved its powerful for selecting features. Senan et al. [27] proposed a method to select the relevant features using RFE algorithm for a kidney disease dataset. Four classification algorithms are applied for the classification step. The RF algorithm gave the best results.

Many researchers used a hybrid method which combined filter and wrapper methods to select relevant features, but it had many limitations that filter method may cancel important features and wrapper methods take more time. High dimensional is another limitation when applying this hybrid [28]. Ansari et al. [10] used filter and wrapper approaches as a feature selection process. They proposed two different hybrid methods. F-score feature ranker and Chi-square feature ranker are applied in the first method and took the intersection between them. The intersection between these features is applied to obtain the most important features. The results of the intersection process are applied on binary particle swarm optimization (BPSO) as a feature optimization approach. In the second one, after the intersection between features, RFE approach is applied. Zhang et al. [7] proposed a method to classify six human cancer types using CNV level values. Zhang selected the features using the methods of mRMR (minimum Redundancy Maximum Relevance Feature selection) and IFS (Incremental Feature Selection). The first method selected features by ranking the importance of these features. This method selected 200 features. The second method used IFS to select the optimal set of features. IFS selected 19 features with an accuracy value 0.75. However, this proposed method gave insufficient classification accuracy. Pirgazi et al. [29] proposed a hybrid method using filter and wrapper for feature selection in high dimensional datasets. In the first stage, they applied a filter method using the Relief method to weight the features. In the second stage, they applied a wrapper method using shuffled frog leaping algorithm (SFLA) and IWSSr algorithms. Mandal et al. [30] proposed a hybrid method for feature selection using the filter and wrapper method. They applied MIFS, ReliefF, Chi-Square, and Xvariance for the filter method. The union for four filter methods is applied to obtain the most important features. The wrapper method is applied using Whale Optimization Algorithm to overcome any limitation in the filter method. Venkatesh et al. [31] proposed a hybrid method using MIFS as a filter method and RFE as a wrapper method. The hybrid method gave better results than the individual algorithms. Gakii et al. [32] proposed comparison methods using three algorithms for feature selection included in the PCA, RFE and graph-based feature selection. The results proved that the graph-based feature selection enhanced the performance of sequential minimal optimization and multilayer perceptron classifiers. In addition, researchers applied a hybrid method using the advantages of both wrapper and embedded methods to obtain the most effective features to solve the drawbacks in the previous studies. Liu et al. [28] proposed a hybrid method using GA as a global search with an embedded regularization approach as a local search. They proposed this method to solve the over-fitting problems and select relevant features. It is compared with individual algorithms, proving its effectiveness for feature selection. Aruna et al. [33] proposed a hybrid method using LR and RFE algorithms for the diabetes dataset. The RFE is based on LR as an estimator. The RF is applied for a classification step. Venkatachalam et al. [34] proposed a hybrid method that combined the ridge regression and RFE algorithms. It solved the problem of over-fitting for feature selection. The proposed method is compared with other models. RF is applied for the classification step.

Due to the previous research gaps, this paper presents the proposed method PFBS-RFS-RFE with three positions to fix feature selection problems and improve the classification model over different datasets. It tries to enhance many issues included in time consuming using RFE algorithm, classification accuracy, over-fitting problems, fitting time and select the most effective features to know the chromosome that is considered the most developing human cancers in the datasets. Furthermore, we applied a resampling method to enhance the classification accuracy and improve the over-fitting problem [35]. The bootstrap is a resampling method that reduces the variance and bias between features; therefore, the over-fitting problem is minimized, and classification accuracy is maximized. We utilize PFBS as a resampling step with the hybrid RFS-RFE to reduce the over-fitting problem and improve the classification accuracy. We compared the proposed methods with RFE, RFS, and with previous work over five datasets. Four efficient supervised machine learning were used to evaluate the model performance of the proposed hybrid feature selection methods. The main contributions are summarized as follows: -

1. We propose hybrid methods, namely, positions first bootstrap step random forest selection recursive feature elimination (PFBS-RFS-RFE) based on feature selection that combines the advantages of the wrapper and embedded methods to solve many feature selection problems, including over-fitting, time consuming, relevant features, classification accuracy and solving the problem in RFE algorithm, which suffers from time-consuming with high-dimensional datasets.
2. The motivation behind the proposed methods is to know the genes or features associated with cancers; therefore, we can know the chromosome that is considered the most developing human cancers by taking the average number of runs and the intersection between features.

The structure of the article is as follows. The “Introduction” section presents the feature selection troubles and how previous work tried to solve them. The “Results” section presents the results of hybrid algorithm and the comparison with other studies using the same datasets. The “Discussion” section summarizes and discusses the application of the hybrid algorithm. The “Conclusions” section presents the main idea and the importance of the proposed methods. The “Method” section presents the hybrid algorithm to enhance and solve these troubles.

## Results

The hybrid proposed methods applied two important stages included in feature selection and model performance. They are applied using proposed datasets to select the effective cancer genes and improve the drawbacks included in over-fitting and classification accuracy. The selected features are utilized to feed more than one classifier using 10 cross-validations. The proposed classifiers are LR, support vector machine (SVM), RF and bagging (Bagg). The proposed method is compared with the individual algorithm such as RFE and RF and with the previous work. The proposed methods confirmed the results.

### Performance metrics

Performance evaluation is a very important step in machine learning. Selecting the most relevant features increases the classification accuracy and decreases the classification error. We proposed a hybrid method to obtain the accurate classification value, therefore; we fixed any previous drawbacks. The proposed methods are compared with individual algorithms included in RFE and RFS using the following metrics: -

- The size of feature selection: - is the number of selected features.
- Processing time: - is the time of the fitting process in second.
- Performance accuracy is the percentage of the samples that are correctly evaluated by a classifier.
- Performance evaluation included: - Precision, F1-score, Recall, variance, Receiver operating characteristic (ROC) area, and Area under curve (AUC) [8, 12] is used to measure the classification performance by plotting the relationship between True Positive (TP) and False Positive (FP) rates.
- The calculation formula is applied to evaluate the model performance using ensemble and regularization classifiers with 10 cross-validation. Table 1 presents the meanings of the symbols that used in the proposed methods. The calculation formula is as follows: -

**Table 1:** The meanings of the symbol

Symbol	Meaning
PPV	Positive predictive value
TP	Tue positive (cancer type diagnosed correctly as a cancer type)
TN	True negative (non-cancer type diagnosed correctly non-cancer type)
FN	False-negative (cancer type diagnosed incorrectly as non-cancer type)
FP	False-positive (non-cancer type diagnosed incorrectly as a cancer type
SF	The size of the selected features after applying the algorithm
TF	The total size of features

1 $$\mathrm{Precision}\ \left(\mathrm{PPV}\right)=\frac{\mathrm{TP}}{\mathrm{TP}+\mathrm{FP}}$$

2 $$\mathrm{Recall}\ \left(\mathrm{Sensitivity}\right)=\frac{\mathrm{TP}}{\mathrm{TP}+\mathrm{FN}}$$

3 $$\mathrm{F}1\text-\mathrm{Score}=\frac{2\ast \mathrm{Precision}\ast \mathrm{Recall}\ }{\mathrm{Precision}+\mathrm{Recall}\ }$$

4 $$\mathrm{ACC}\ \left(\mathrm{Accuracy}\right)=\frac{\mathrm{TP}+\mathrm{TN}}{\mathrm{TP}+\mathrm{TN}+\mathrm{FN}+\mathrm{FP}}$$

### Parameter setting

The experiments were run in Python on a pc with windows 10, R TM CPU 1.80 GHz, and 8 GB memory. All parameter values are determined based on domain-specific knowledge or trial and error. The parameter setting for all proposed methods is given in Table 2, with a simple declaration for each parameter.

**Table 2:** The meaning of parameter setting

Parameter	Value	Definition
NRuns	20	No of runs
Problem Dimensions	–	No of features in the dataset.
X^*^	2916	The number of data produced after the bootstrap resamples method.
M	100	The number of trees using in the Random Forest algorithm.
Criterion	–	The method which measures the quality of split, Entropy is applied.
min_samples_leaf	100	The minimum number of samples required to be at a leaf node.
RFE estimators	–	A supervised learning algorithm. LR is applied.
C	0.05	Regularization parameter.
Max-iteration	100	Max iteration in LR classifier.
Tol	0.0001	Tolerance to stop criteria in LR classification.
CV	10	No of folds in cross-validation.

### Numerical results and discussion

The fundamental goal of these proposed methods is to enhance the performance of RFE to reach the optimum subset features that show the most associated features (genes) with cancers. Another goal of the proposed methods is to solve and fix the problem of over-fitting between training and testing data. The proposed method was compared with the original algorithms included in RFE and RFS. Table 3 presents the performance of the individual algorithms such as RFE and RF using the proposed classifiers LR with 10 folds stratified cross-validation before applying the feature selection proposed methods. Stratified cross-validation splits data into folds to ensure that the ratio between label classes is the same in each fold as in the full data.

In Table 3, the RFE algorithm spent more time on feature selection with high-dimensional datasets. Therefore, it did not achieve good results for classification accuracy. The Parkinson’s disease dataset shows that the classification accuracy achieved low results before applying the proposed methods. Using the BreastEw dataset, we can notice that both RFE and RFS achieved the best results before applying the proposed methods. Still, we need to reach optimal classification accuracy with the smallest subset features. The terms Algo., over-fitting Diff., Pre, Rec, NO.F, F-Time, C-Time, and var. referred to proposed algorithms, difference percentage between training and testing dataset, Precision, Recall, Number of selected features, Fitting time of feature selection, classification fitting time and variance, respectively.

**Table 3:** Performance of original algorithms before applying the proposed methods

Algo.	Train %	Test %	Over- Fitting Diff. %	Pre	Rec	F1- Score	NO.F	F-Time (sec)	C-Time (sec)	AUC	Var.	ACC %
**RNA gene dataset**												
**RFE**	100.000	99.800	**0.200**	0.999	0.998	0.998	**10,265**	**190,000**	60.000	1.000	0.00002	99.800
**RFS**	100.000	99.800	**0.200**	0.999	0.998	0.998	**374.000**	**13.015**	0.275	1.000	0.00002	99.800
**DNA CNV dataset**												
**RFE**	97.500	87.000	**10.500**	0.741	0.706	0.709	**8190**	**182,295**	40.000	0.960	0.023125	87.000
**RFS**	89.803	84.054	**5.749**	0.819	0.764	**0.775**	**1234**	**5.000**	5.085	0.955	0.000193	84.054
**Parkinson’s disease dataset**												
**RFE**	76.441	75.133	**1.308**	0.384	0.480	**0.426**	**376.000**	144.783	0.177	0.689	0.00145	75.133
**RFS**	76.484	75.000	**1.484**	0.629	0.557	**0.537**	**224.000**	**1.474**	0.158	0.706	0.00108	75.000
**BreastEW dataset**												
**RFE**	95.000	94.000	**1.000**	0.948	0.937	0.941	**15.000**	0.142	0.099	**0.990**	**0.00050**	94.000
**RFS**	95.000	93.000	2.000	0.938	0.928	0.932	27.000	0.090	0.008	0.989	0.00583	93.000
**Dermatology erythemato-squamous diseases dataset**												
**RFE**	97.541	96.997	**0.544**	0.972	0.960	0.963	**17.000**	0.062	0.001	0.997	0.001073	96.997
**RFS**	89.860	87.725	2.135	0.837	0.821	0.815	**11.000**	0.094	0.002	0.985	0.002819	87.725

We noticed the previous results that the single algorithms suffered from many problems in the fitting time of feature selection (F-Time), classification fitting time (C-Time), number of selected features, over-fitting, and classification accuracy. Therefore, we proposed the methods to fix any previous problems in original algorithms when run as a single algorithm and obtain the most effective cancers genes. In addition, we noticed that the single algorithms did not give the best results, so we applied a hybrid method using the wrapper and embedded procedure.

In Table 4, the average results of the proposed method OFBS-RFS-RFE are presented using stratified cross-validation with proposed classifiers included in LR, SVM, RF and Bagg. The proposed methods are run 2o times to obtain the best results. The PFBS has many positions of the first bootstrap step included in OFBS, IFBS and both outer and O/IFBS. The following table presented the OFBS-RFSRFE after 20 runs.

**Table 4:** Average results after applying OFBS-RFS-RFE after 20 runs

Algo.	Train Data %	Test Data %	Over-fitting Diff. %	Pre	Rec	F1-score	No.F	F-Time (sec)	C-Time (sec)	AUC	Var.	ACC %
**RNA gene dataset**												
**LR classifier**												
**OFBS-RFS**	100.000	99.944	0.056	1.000	0.999	1.000	379.100	9.537	0.296	1.000	0.000003	99.944
**OFBS-RFS-RFE**	100.000	99.981	0.019	1.000	1.000	1.000	142.500	189.35	0.445	1.000	**0.0000004**	**99.981**
**SVM classifier**												
**OFBS-RFS**	100.000	99.945	0.055	1.000	0.999	1.000	379.100	9.537	0.296	1.000	0.000003	99.945
**OFBS-RFS-RFE**	100.000	95.038	4.962	1.000	0.961	1.000	142.500	189.35	0.192	1.000	**0.0000002**	95.038
**RF classifier**												
**OFBS-RFS**	100.000	99.875	0.125	0.999	0.999	0.999	379.100	9.537	1.007	0.999	0.000013	99.875
**OFBS-RFS-RFE**	100.000	99.925	0.075	1.000	0.999	0.999	142.500	189.35	0.807	0.999	0.000005	99.925
**Bagg classifier**												
**OFBS-RFS**	99.967	99.439	0.528	0.995	0.994	0.994	379.100	9.537	0.912	0.999	0.000074	99.439
**OFBS-RFS-RFE**	99.972	99.513	0.459	0.996	0.995	0.995	142.500	189.35	0.4482	0.999	0.000063	99.513
**DNA CNV dataset**												
**LR classifier**												
**OFBS-RFS**	93.838	90.857	2.981	0.914	0.875	0.888	**1351**	5.435	5.620	0.981	0.00138	90.857
**OFBS-RFS-RFE**	93.462	**91.020**	**2.442**	**0.919**	0.875	0.887	**675.000**	2755	**2.637**	**0.983**	**0.00028**	**91.020**
**SVM classifier**												
**OFBS-RFS**	94.248	90.980	3.268	0.923	0.873	0.890	**1351**	5.435	27.316	0.981	0.00012	90.980
**OFBS-RFS-RFE**	94.248	90.980	3.268	0.923	0.873	0.890	**675.000**	2755	27.316	0.981	0.00048	90.980
**RF classifier**												
**OFBS-RFS**	90.966	86.613	4.353	0.918	0.834	0.888	**1351**	5.435	2.934	0.985	0.00021	86.613
**OFBS-RFS-RFE**	**95.687**	**91.265**	4.421	0.921	0.875	0.890	**675.000**	2755	2.147	0.986	0.00074	91.265
**Bagg classifier**												
**OFBS-RFS**	98.622	92.971	5.651	0.927	0.907	0.916	**1351**	5.435	9.080	0.929	0.00024	92.971
**OFBS-RFS-RFE**	**95.525**	**92.762**	**2.763**	**0.925**	**0.910**	**0.912**	**675.000**	2755	4.502	**0.981**	**0.00023**	**92.762**
**Parkinson’s disease dataset**												
**LR classifier**												
**OFBS-RFS**	78.453	77.864	0.589	0.737	0.602	0.605	228.150	1.704	0.128	0.736	0.00103	77.864
**OFBS-RFS-RFE**	77.050	72.740	4.310	0.700	0.556	0.579	113.850	11.213	0.149	0.705	0.00093	72.740
**SVM classifier**												
**OFBS-RFS**	76.109	75.624	0.485	0.623	0.543	0.512	228.150	1.704	0.644	0.643	0.00043	75.624
**OFBS-RFS-RFE**	76.003	75.499	0.504	0.617	0.541	0.509	113.850	11.214	0.496	0.638	0.00041	75.499
**RF classifier**												
**OFBS-RFS**	**100.000**	94.634	**5.366**	0.948	0.910	0.926	228.150	1.704	1.434	0.986	0.00064	94.634
**OFBS-RFS-RFE**	**100.000**	95.000	**5.000**	**0.945**	**0.906**	**0.922**	**113.850**	**11.214**	**1.134**	**0.985**	**0.00062**	**95.000**
**Bagg classifier**												
**OFBS-RFS**	99.719	93.163	6.556	0.917	0.904	0.908	228.150	1.704	1.790	0.966	0.00092	93.163
**OFBS-RFS-RFE**	99.735	93.008	6.727	0.916	0.901	0.906	113.850	11.214	0.820	0.966	0.00078	93.008
**Dermatology erythemato-squamous diseases dataset**												
**LR classifier**												
**OFBS-RFS**	96.023	95.037	0.986	0.926	0.912	0.907	18.625	0.216	0.014	0.995	0.000190	95.037
**OFBS-RFS-RFE**	97.241	96.481	0.760	0.932	0.934	0.926	16.000	0.203	0.517	0.997	0.000730	96.481
**SVM classifier**												
**OFBS-RFS**	79.269	78.375	0.894	0.672	0.708	0.668	18.625	0.216	0.169	0.973	0.002590	78.375
**OFBS-RFS-RFE**	99.484	98.940	0.544	0.988	0.986	0.984	16.000	0.203	0.064	0.998	0.000368	98.940
**RF classifier**												
**OFBS-RFS**	**100.000**	**100.000**	**0.0**	**1.000**	**1.000**	**1.000**	**18.625**	**0.216**	**0.562**	**1.000**	**0.0**	**100.000**
**OFBS-RFS-RFE**	**100.000**	**100.000**	**0.0**	**1.000**	**1.000**	**1.000**	**16.000**	**0.203**	**0.500**	**1.000**	**0.0**	**100.000**
**Bagg classifier**												
**OFBS-RFS**	99.970	99.730	0.240	0.997	0.995	0.996	18.625	0.216	0.077	0.997	0.000057	99.730
**OFBS-RFS-RFE**	99.966	99.796	0.170	0.998	0.995	0.996	16.000	0.203	0.242	0.998	0.000055	99.796
**BreastEW dataset**												
**LR classifier**												
**OFBS-RFS**	94.776	94.218	0.558	0.922	0.933	0.937	27.100	0.298	**0.012**	0.988	0.000001	94.218
**OFBS-RFS-RFE**	95.069	94.587	0.482	0.947	0.939	0.941	13.316	0.130	**0.091**	0.989	0.000810	94.587
**SVM classifier**												
**OFBS-RFS**	92.167	91.902	0.266	0.934	0.897	0.909	27.100	0.298	0.076	0.978	0.000986	91.902
**OFBS-RFS-RFE**	93.301	93.114	0.187	0.913	0.914	0.927	13.316	0.130	**0.070**	0.982	0.001115	93.114
**RF classifier**												
**OFBS-RFS**	100.000	97.864	2.136	0.984	0.981	0.979	27.100	0.298	0.506	0.997	0.000270	97.864
**OFBS-RFS-RFE**	100.000	98.000	**2.000**	0.983	0.979	0.982	13.316	0.130	0.428	**0.997**	**0.000300**	**98.000**
**Bagg classifier**												
**OFBS-RFS**	99.889	97.548	2.341	0.977	0.972	0.974	27.100	0.298	0.101	0.949	0.000280	97.548
**OFBS-RFS-RFE**	99.888	97.724	2.164	0.978	0.974	0.976	13.316	0.130	0.104	0.948	0.000430	97.724

For more illustration, in Table 4, the proposed method using OFBS-RFS-RFE enhanced the performance of RFE algorithm. The over-fitting percentage was reduced from the RNA gene dataset after applying previous classifiers, so the accuracy difference between training and testing dataset was reduced compared with the single algorithm. The LR classifier achieved the best classification accuracy result with 99.981%, while the SVM classifier gave the best variance result with 0.0000002. From DNA CNV dataset the difference between training and testing became 2.442 and 2.763% using LR and Bagg classifiers, respectively, and the accuracy results were increased with 91.020 and 92.762%, respectively using the same classifiers. In addition, the variance between features was reduced using the same classifiers to become 0.00028 and 0.00023, respectively. The OFBS-RFS-RFE enhances the over-fitting and variance and minimizes features’ fitting time and number. From the Parkinson’s disease dataset, the classification accuracy, precision, recall, f1-score, AUC and variance are enhanced to 95.000%, 0.945, 0.906, 0.922, 0.985 and 0.00062, respectively using RF classifier. It suggested that only 113.85 features were good enough for the classification step with 1.134 s as a computational time. In addition, for dermatology erythemato-squamous diseases dataset, RF classifier gave the best classification accuracy, precision, recall, f1-score, AUC and variance to become 100.000%, 1.000, 1.000, 1.000, 1.000 and 0.0. On the other hand, the OFBS-RFS-RFE using the BreastEw dataset achieved the best computational time after applying LR and SVM in contrast with the other optimizer. We can notice that the RF gave the best over-fitting percentage, precision, recall, f1-score, AUC, variance, and accuracy to become 2.00%, 0.983, .979, 0.982, 0.997, 0.000302 and 98%, respectively.

In Table 5, the average results of the proposed method PFBS-RFS-RFE using IFBS after 20 runs are presented. The different positions of bootstrap lead to different results. The IFBS used the bootstrap step inside the RFS algorithm for feature selection.

**Table 5:** Average results after applying IFBS-RFS-RFE after 20 runs

Algo.	Train Data %	Test Data %	Over-fitting Diff. %	Pre	Rec	F1-score	No.F	F-Time (sec)	C-Time (sec)	AUC	Var.	ACC %
**RNA gene dataset**												
**LR Classifier**												
**IFBS-RFS**	100.000	99.925	0.075	0.999	0.999	0.999	239.000	5.421	0.193	1.000	0.000004	99.925
**IFBS-RFS-RFE**	100.000	99.975	0.025	0.999	0.999	0.999	125.250	15.201	0.357	1.000	**0.0000004**	**99.975**
**SVM Classifier**												
**IFBS-RFS**	99.999	99.906	0.093	0.999	0.998	0.999	239.000	5.421	0.225	1.000	0.000005	99.906
**IFBS-RFS-RFE**	100.000	99.988	0.012	0.999	0.999	0.999	125.250	15.201	0.153	1.000	**0.0000002**	**99.988**
**RF Classifier**												
**IFBS-RFS**	100.000	99.694	0.306	0.998	0.997	0.997	239.000	5.421	0.901	1.000	0.000030	99.694
**IFBS-RFS-RFE**	100.000	99.807	0.193	0.999	0.998	0.998	125.250	15.201	0.737	0.999	0.000019	99.807
**Bagg Classifier**												
**IFBS-RFS**	99.947	99.002	0.945	0.991	0.989	0.989	239.000	5.421	0.635	0.999	0.000075	99.002
**IFBS-RFS-RFE**	99.955	99.027	0.928	0.992	0.989	0.990	125.250	15.201	0.327	0.999	0.000075	99.027
**DNA CNV dataset**												
**LR Classifier**												
**IFBS-RFS**	88.525	82.889	5.636	0.812	0.752	0.763	966.000	6.250	3.876	0.942	0.000370	82.889
**IFBS-RFS-RFE**	88.000	84.000	**4.000**	0.831	0.764	0.804	482.000	1545	**2.149**	0.959	**0.000420**	**84.000**
**SVM Classifier**												
**IFBS-RFS**	88.341	81.637	6.704	0.815	0.729	0.745	966.000	6.050	29.078	0.955	0.000580	81.637
**IFBS-RFS-RFE**	89.668	82.268	7.400	0.827	0.738	0.753	482.000	1545	15.721	0.960	0.000880	82.268
**RF Classifier**												
**IFBS-RFS**	89.660	80.089	9.571	0.768	0.7025	0.709	966.000	6.050	3.150	0.938	0.000410	80.089
**IFBS-RFS-RFE**	89.935	80.138	9.797	0.770	0.703	0.719	482.000	1545	2.487	0.941	0.000470	80.138
**Bagg Classifier**												
**IFBS-RFS**	97.697	78.316	19.381	0.733	0.722	0.702	966.000	6.050	7.850	0.867	0.000450	78.316
**IFBS-RFS-RFE**	89.035	78.309	10.726	0.730	0.695	0.702	482.000	1545	4.023	0.910	0.000480	78.309
**Parkinson’s disease dataset**												
**LR Classifier**												
**IFBS-RFS**	78.122	76.718	1.404	0.664	0.588	0.582	154.263	1.071	0.082	0.732	0.002210	76.718
**IFBS-RFS-RFE**	76.693	73.998	2.695	0.667	0.588	0.581	80.050	4.594	0.164	0.722	0.001990	73.998
**SVM Classifier**												
**IFBS-RFS**	75.684	72.248	3.436	0.468	0.498	0.448	154.263	1.071	0.482	0.621	0.000770	72.248
**IFBS-RFS-RFE**	75.697	72.228	3.469	0.464	0.497	0.448	80.050	4.594	0.407	0.619	0.000760	72.228
**RF Classifier**												
**IFBS-RFS**	99.999	83.912	16.087	0.811	0.738	0.760	154.263	1.071	1.230	0.866	0.003480	83.912
**IFBS-RFS-RFE**	100.000	81.485	18.515	0.773	0.700	0.719	80.050	4.594	0.964	0.834	0.003500	81.485
**Bagg Classifier**												
**IFBS-RFS**	99.590	80.810	17.78	0.754	0.731	0.737	154.263	1.071	1.225	0.826	0.003460	80.810
**IFBS-RFS-RFE**	99.580	79.191	20.389	0.729	0.707	0.713	80.050	4.594	0.546	0.804	0.003440	79.191
**Dermatology erythemato-squamous diseases dataset**												
**LR classifier**												
**IFBS-RFS**	91.531	91.000	0.531	0.771	0.796	0.777	13.000	0.515	0.002	0.988	0.000881	91.000
**IFBS-RFS-RFE**	92.198	91.801	0.397	0.773	0.799	0.780	12.000	0.016	0.020	0.988	0.000975	91.801
**SVM classifier**												
**IFBS-RFS**	94.870	93.979	0.891	0.888	0.878	0.875	13.000	0.515	0.023	0.988	0.001285	93.979
**IFBS-RFS-RFE**	94.869	93.979	0.890	0.888	0.878	0.875	12.000	0.016	0.075	0.989	0.001285	93.979
**RF classifier**												
**IFBS-RFS**	97.025	93.183	3.482	0.900	0.892	0.889	13.000	0.515	0.142	0.984	0.001493	93.183
**IFBS-RFS-RFE**	97.000	93.500	3.500	0.900	0.892	0.889	12.000	0.016	0.140	0.980	0.001490	93.500
**Bagg classifier**												
**IFBS-RFS**	96.903	92.102	4.801	0.895	0.884	0.881	13.000	0.515	0.016	0.989	0.003297	92.102
**IFBS-RFS-RFE**	97.177	81.194	15.983	0.789	0.764	0.760	12.000	0.016	0.014	0.970	0.081251	81.194
**BreastEW dataset**												
**LR Classifier**												
**IFBS-RFS**	94.394	93.678	0.461	0.938	0.938	0.938	23.100	0.410	0.012	0.988	0.000690	93.678
**IFBS-RFS-RFE**	94.855	94.403	**0.452**	0.946	0.946	0.946	11.900	0.103	0.091	0.992	0.000520	94.403
**SVM Classifier**												
**IFBS-RFS**	92.010	91.563	0.447	0.929	0.929	0.929	23.100	0.410	0.069	0.976	0.001010	91.563
**IFBS-RFS-RFE**	93.888	93.503	0.385	0.944	0.944	0.944	11.900	0.103	0.059	0.983	0.000550	93.503
**RF Classifier**												
**IFBS-RFS**	100.000	96.411	3.589	0.965	0.965	0.965	23.100	0.410	0.452	0.991	0.000980	96.411
**IFBS-RFS-RFE**	100.000	95.277	4.723	0.952	0.952	0.952	11.900	0.103	0.433	0.989	0.000930	95.277
**Bagg Classifier**												
**IFBS-RFS**	99.625	95.302	4.323	0.954	0.954	0.954	23.100	0.410	0.099	0.985	0.000920	95.302
**IFBS-RFS-RFE**	99.610	94.416	5.194	0.944	0.944	0.944	11.900	0.103	0.085	0.981	0.001170	94.416

For more illustration, in Table 5, the SVM classifier achieved the best classification accuracy and variance results with 99.988% and 0.0000002, respectively. Although the inner position gave the best results using RNA gene dataset, but it did not give the best result for other datasets.

In Table 6, the average results of PFBS-RFS-RFE using O/IFBS after 20 runs are presented. In this position the FBS is placed before selecting the features and during the feature selecting algorithm.

**Table 6:** Average results after applying O/IFBS-RFS-RFE after 20 runs

Algo.	Train Data %	Test Data %	Over-fitting Diff. %	Pre	Rec	F1-score	NO.F	F-Time (sec)	C-Time (sec)	AUC	Var.	ACC %
**RNA gene dataset**												
**LR Classifier**												
**O/IFBS-RFS-**	100.000	99.975	0.025	0.999	0.999	0.999	238.800	4.220	0.176	1.000	0.0000006	99.975
**O/IFBS-RFS-RFE**	100.000	99.994	0.006	0.999	0.999	0.999	119.200	13.726	0.307	1.000	0.0000004	**99.994**
**SVM Classifier**												
**O/IFBS-RFS-**	100.000	99.950	0.05	0.999	0.999	0.999	238.800	4.220	0.197	1.000	0.0000025	99.950
**O/IFBS-RFS-RFE**	100.000	99.981	0.019	0.999	0.999	0.999	119.200	13.726	0.125	1.000	0.0000004	99.981
**RF Classifier**												
**O/IFBS-RFS-**	100.000	99.888	0.112	0.999	0.999	0.999	238.800	4.220	0.755	1.000	0.0000076	99.888
**O/IFBS-RFS-RFE**	100.000	99.913	0.087	0.999	0.999	0.999	119.200	13.726	0.596	0.999	0.0000054	99.913
**Bagg Classifier**												
**O/IFBS-RFS-**	99.974	99.357	0.617	0.994	0.992	0.993	238.800	4.220	0.513	0.999	0.0000828	99.357
**O/IFBS-RFS-RFE**	99.972	99.363	0.609	0.994	0.993	0.993	119.200	13.726	0.266	0.999	0.000083	99.363
**DNA CNV dataset**												
**LR Classifier**												
**O/IFBS-RFS-**	92.581	89.818	2.763	0.904	0.861	0.877	973.000	3.650	3.850	0.975	0.00031	89.818
**O/IFBS-RFS-RFE**	91.878	89.601	2.277	0.906	0.857	0.885	485.000	1460	1.950	0.936	0.00035	89.601
**SVM Classifier**												
**O/IFBS-RFS-**	93.361	90.253	3.108	0.917	0.860	0.878	973.000	3.650	22.00	0.980	0.00065	90.253
**O/IFBS-RFS-RFE**	94.241	90.979	3.262	0.925	0.873	0.891	485.000	1460	11.700	0.985	0.00028	90.979
**RF Classifier**												
**O/IFBS-RFS-**	95.527	90.764	4.763	0.914	0.868	0.882	973.000	3.650	2.650	0.984	0.00027	90.764
**O/IFBS-RFS-RFE**	95.681	90.954	4.727	0.919	0.872	0.890	485.000	1460	1.750	0.941	0.00027	90.954
**Bagg Classifier**												
**O/IFBS-RFS-**	97.958	92.712	5.246	0.926	0.906	0.913	973.000	3.650	6.550	0.980	0.00027	92.712
**O/IFBS-RFS-RFE**	**95.318**	**92.834**	**2.484**	**0.927**	**0.906**	**0.913**	**485.000**	1460	3.150	**0.980**	**0.00027**	**92.834**
**Parkinson’s disease dataset**												
**LR classifier**												
**O/IFBS-RFS-**	79.050	78.482	0.568	0.742	0.619	0.626	155.50	1.058	0.093	0.764	0.00123	78.482
**O/IFBS-RFS-RFE**	77.744	77.427	0.317	0.712	0.597	0.598	77.550	5.551	0.118	0.731	0.00092	77.427
**SVM classifier**												
**O/IFBS-RFS-**	76.009	75.442	0.567	0.612	0.539	0.508	155.500	1.058	0.511	0.637	0.00041	75.442
**O/IFBS-RFS-RFE**	77.500	76.672	0.828	0.653	0.566	0.542	77.550	5.551	0.420	0.669	0.00051	76.672
**RF classifier**												
**O/IFBS-RFS-**	100.000	94.494	5.506	0.945	0.909	0.924	155.500	1.058	1.122	0.985	0.00064	94.494
**O/IFBS-RFS-RFE**	**100.000**	94.082	**5.918**	0.943	0.901	0.917	77.550	5.551	0.911	0.983	0.00070	**94.082**
**Bagg Classifier**												
**O/IFBS-RFS-**	99.720	93.196	**6.524**	0.916	0.906	0.909	155.500	1.058	1.091	0.965	0.00093	**93.196**
**O/IFBS-RFS-RFE**	99.719	92.917	**6.802**	0.914	0.900	0.905	77.550	5.550	0.511	0.966	0.00084	**92.917**
**Dermatology erythemato-squamous diseases dataset**												
**LR classifier**												
**O/IFBS-RFS-**	96.691	96.441	0.250	0.649	0.624	0.630	11.000	0.167	0.025	0.998	0.000848	96.441
**O/IFBS-RFS-RFE**	92.532	92.350	0.212	0.801	0.751	0.766	10.000	0.500	0.128	0.999	0.000790	92.350
**SVM classifier**												
**O/IFBS-RFS-**	95.082	95.000	0.082	0.638	0.608	0.613	11.000	0.167	0.025	0.977	0.000632	95.000
**O/IFBS-RFS-RFE**	98.361	98.356	0.005	0.892	0.900	0.895	10.000	0.500	0.047	0.999	0.001040	98.356
**RF classifier**												
**O/IFBS-RFS-**	**100.000**	**100.000**	**0.0**	**1.000**	**1.000**	**1.000**	**11.000**	**0.167**	**0.562**	**1.000**	**0.0**	**100.00**
**O/IFBS-RFS-RFE**	**100.000**	**100.000**	**0.0**	**1.000**	**1.000**	**1.000**	**10.000**	0.500	0.500	**1.000**	**0.0**	**100.00**
**Bagg classifier**												
**O/IFBS-RFS-**	**100.000**	**100.000**	**0.0**	**1.000**	**1.000**	**1.000**	**11.000**	0.167	0.520	0.999	0.0	**100.000**
**O/IFBS-RFS-RFE**	**100.000**	**100.000**	**0.0**	**1.000**	**1.000**	**1.000**	**10.000**	0.500	0.500	0.991	0.0	**100.000**
**BreastEw dataset**												
**LR classifier**												
**O/IFBS-RFS-**	94.647	94.148	0.499	0.944	0.932	0.936	22.900	0.399	0.010	0.988	0.00095	94.148
**O/IFBS-RFS-RFE**	95.305	94.842	0.463	0.949	0.942	0.944	11.300	0.1033	0.091	0.992	0.00086	94.842
**SVM classifier**												
**O/IFBS-RFS-**	92.110	91.889	0.221	0.934	0.897	0.909	22.900	0.399	0.067	0.978	0.00098	91.889
**O/IFBS-RFS-RFE**	93.515	93.400	0.115	0.943	0.918	0.927	11.300	0.103	0.058	0.983	0.00094	93.400
**RF classifier**												
**O/IFBS-RFS-**	99.563	97.500	2.063	0.981	0.976	0.977	22.900	0.3994	0.411	0.996	0.00031	97.500
**O/IFBS-RFS-RFE**	**100.000**	**98.000**	2.000	0.979	0.977	0.978	11.300	0.103	0.404	0.997	0.00031	**98.000**
**Bagg Classifier**												
**O/IFBS-RFS-**	99.819	97.618	2.201	0.977	0.973	0.974	22.900	0.399	0.089	0.994	0.00038	97.618
**O/IFBS-RFS-RFE**	99.803	97.505	2.298	0.976	0.972	0.973	11.300	0.103	0.065	0.993	0.00034	97.505

For more illustration, in Table 6, the accuracy and variance results are increased from the RNA gene dataset to 99.994% and 0.0000004, respectively, using LR classifier. Bagg classifier gave the best accuracy and variance results using DNA CNV dataset to become 92.834% and 0.00027, respectively. In addition, RF classifier gave the best accuracy and variance using dermatology erythemato-squamous diseases dataset to become 100% and 0.0, respectively. At the same time, the O/IFBS-RFS-RFE did not give good results for other datasets.

**Fig. 1:**
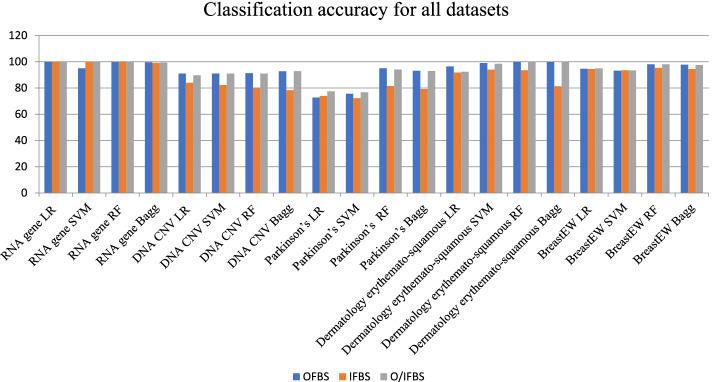
Comparison between proposed methods on all datasets using classification accuracy

In Fig. 1, the classification accuracy using the proposed methods is illustrated using all datasets. We can notice that RNA gene dataset achieved the best results with O/IFBS using LR classifier, while the DNA CNV dataset achieved the best results with O/IFBS using Bagg classifier. In addition, the Parkinson’s disease dataset achieved the best results with OFBS using LR classifier. The dermatology erythemato-squamous diseases and breast datasets achieved the best result using RF classifier with both OFBS and O/IFBS.

**Fig. 2:**
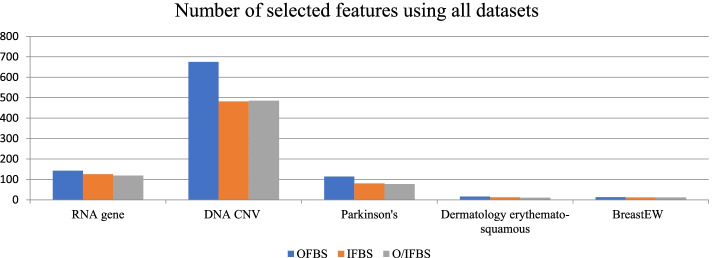
Number of the selected features using all datasets

In Fig. 2, the number of selected features using the proposed methods is showed on all datasets. From this figure, we can note that the best algorithm that gave the smallest number of features was O/IFBS with RNA gene, Parkinson’s disease, dermatology erythemato-squamous diseases and breast datasets. On the other hand, the IFBS algorithm achieved the smallest number of features using DNA CNV dataset.

**Fig. 3:**
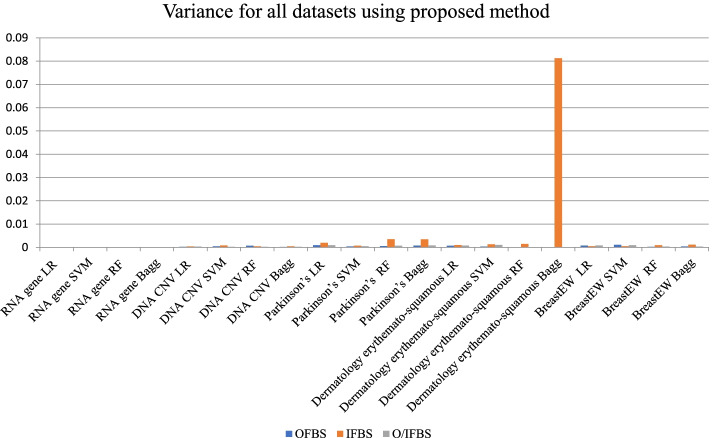
Variance of the proposed methods using all bootstrap positions

In Fig. 3, the variance of the proposed methods is illustrated. We can notice that the RNA gene dataset using LR and SVM classifiers gave the best variance results with all position of bootstrap. On the other hand, the DNA CNV dataset achieved the best variance result using the Bagg classifier with OFBS. In addition, the Parkinson’s disease dataset achieved the best variance result using SVM classifier with OFBS. OFBS and O/IFBS achieved the best variance result using RF and Bagg classifiers for dermatology erythemato-squamous diseases dataset. For Breast dataset, the RF classifier gave the best results with OFBS.

### Comparison with other studies

The results before and after PFBS-RFS-RFE are compared. In addition, these results are compared with the previous work using the same datasets. Table 7 showed the comparison before and after applying PFBS-RFS-RFE after 20 runs. The proposed methods improved the results and solved feature selection problems in high dimensions. Table 8 presented the results of the previous studies using the same dataset.

**Table 7:** The comparison between results before and after PFBS-RFS-RFE

Datasets	Before PFBS-RFS-RFE					After PFBS-RFS-RFE				
	ACC %	Overfitting Diff%	No.F	C-time	Var.	ACC %	Overfitting Diff%	NO. F	C-time	Var.
RNA gene	99.800	0.200	20,531	16.547	0.000015	99.994	0.006	119.200	0.307 s	0.0000004
DNA CNV	85.000	12.600	16,381	170 s	0.000580	92.762	2.763	675.000	0.981 s	0.000230
Parkinson’s disease	93.677	0.800	753	2.000 s	0.000634	95.000	5.000	113.850	1.134 s	0.000620
Dermatology diseases	97.807	0.493	34	0.003 s	0.000810	**100.000**	**0.0**	**10.000**	**0.500 s**	**0.0**
BreastEW	75.928	2.072	30	0.500 s	0.002092	98.000	2.000	13.300	0.428 s	0.000300

**Table 8:** Achievement of accuracy in different research for cancer classification using the same datasets [7–9, 12, 36, 37]

Reference	Dataset	FS Approach	No of selected features	Var.	AUC	ACC %
García-Díaz et al. [36]	RNA gene	GGA	49	0.000303	–	98.810
Zhang et al. [7]	DNA CNV	mRMR & IFS	19	0.000580	0.973	75.000
Sanaa et al. [8]		PSO & GA	2050	–	0.961	84.600
Sanaa et al. [12]		IG	16,381	–	0.965	85.900
Sakar et al. [37]	Parkinson’s disease	,mRMR	50	–	–	85.000
Hegazy et al. [9]	BreastEw	CSSA	5.200	–	–	97.080

The proposed methods were compared with filter ones methods using MIFS, IGF and mRMR. Tables 9, 10 and 11 showed the results of MIFS, IGF and mRMR for all datasets. For MIFS method, the results proved that the LR classifier gave the best accuracy for RNA gene and DNA CNV datasets, while the RF classifier gave the best accuracy for Parkinson’s disease and BreastEW datasets. In addition, SVM classifier gave the best results for dermatology erythemato-squamous diseases dataset. For IGF method, LR classifier gave the best accuracy for RNA gene dataset. SVM classifier gave the best results for DNA CNV and dermatology erythemato-squamous diseases datasets, while the RF classifier gave the best accuracy for Parkinson’s disease and BreastEW datasets. Furthermore, mRMR achieved the best results for RNA gene dataset using LR classifer, while SVM classifier gave the best results for DNA CNV dataset. In addition, RF classifer achieved the best results for dermatology erythemato-squamous diseases, Parkinson’s disease and BreastEW datasets. Although filter ones methods improved the results, they did not give better results than the PFBS-RFS-RFE.

**Table 9:** The proposed methods compared with the MIFS method

Datasets	Train Data %	Test Data %	Over-fitting Diff. %	Pre	Rec	F1-score	NO.F	F-Time (sec)	C-Time (sec)	AUC	Var.	ACC %
**LR classifier**												
RNA gene	100.000	99.875	0.125	0.999	0.998	0.988	10,000	192.552	2.896	1.000	0.000016	99.875
DNA CNV	96.597	84.978	11.619	0.817	0.782	0.788	9000	173.955	25.195	0.954	0.000416	84.978
Parkinson’s disease	77.058	75.525	1.533	0.620	0.556	0.538	300	0.377	0.037	0.682	0.001001	75.525
Dermatology diseases	97.845	96.989	0.856	0.971	0.965	0.966	25	0.203	0.003	0.997	0.000585	96.989
BreastEW	94.396	93.678	0.718	0.938	0.928	0.932	20	0.067	0.002	0.988	0.000694	93.678
**SVM classifier**												
RNA gene	100.000	99.750	0.250	0.998	0.997	0.997	10,000	192.552	2.534	1.000	0.000028	99.750
DNA CNV	91.606	84.122	7.484	0.860	0.756	0.775	9000	173.955	75.394	0.949	0.000668	84.122
Parkinson’s disease	75.676	72.228	3.448	0.472	0.498	0.448	300	0.203	0.138	0.627	0.000814	72.228
Dermatology diseases	98.421	97.523	0.898	0.976	0.967	0.969	25	0.203	0.028	0.998	0.000924	97.523
BreastEW	92.013	91.563	0.450	0.929	0.895	0.906	20	0.067	0.017	0.976	0.001014	91.563
**RF classifier**												
RNA gene	100.000	99.627	0.373	0.998	0.996	0.997	10,000	192.552	1.252	1.000	0.000036	99.627
DNA CNV	92.962	80.623	12.339	0.771	0.719	0.718	9000	173.955	3.528	0.942	0.000614	80.623
Parkinson’s disease	100.000	84.782	15.218	0.827	0.748	0.773	300	0.377	0.376	0.876	0.002303	84.782
Dermatology diseases	100.000	96.456	3.544	0.972	0.950	0.955	25	0.203	0.148	0.999	0.001473	96.456
BreastEW	100.000	96.140	3.860	0.963	0.956	0.958	20	0.067	0.110	0.990	0.000944	96.140
**Bagg classifier**												
RNA gene	99.847	98.628	1.219	0.989	0.985	0.987	10,000	192.552	7.322	0.999	0.000036	98.628
DNA CNV	98.960	78.806	20.154	0.733	0.699	0.707	9000	173.955	25.309	0.912	0.000613	78.806
Parkinson’s disease	99.574	79.239	20.335	0.729	0.729	0.727	300	0.377	0.673	0.794	0.002002	79.239
Dermatology diseases	99.696	95.105	4.591	0.955	0.940	0.939	25	0.203	0.021	0.995	0.001473	95.105
BreastEW	99.492	95.435	4.057	0.957	0.947	0.950	20	0.067	0.022	0.986	.000901	95.435

**Table 10:** The proposed methods compared with the IGF method

Datasets	Train Data %	Test Data %	Over-fitting Diff. %	Pre	Rec	F1-score	NO.F	F-Time (sec)	C-Time (sec)	AUC	Var.	ACC %
**LR classifier**												
RNA gene	100.000	99.875	0.125	0.999	0.999	0.998	3576	1.182	2.121	1.000	0.000016	99.875
DNA CNV	93.115	81.310	11.805	0.782	0.706	0.705	3315	5.651	0.595	0.951	0.000576	81.310
Parkinson’s disease	77.822	76.984	0.838	0.680	0.576	0.566	396	0.093	0.057	0.710	0.001445	76.984
Dermatology diseases	97.784	97.260	0.524	0.973	0.968	0.969	25	0.032	0.0009	0.998	0.000677	97.260
BreastEW	94.170	93.674	0.496	0.942	0.928	0.931	22	0.064	0.001	0.989	0.002176	93.674
**SVM classifier**												
RNA gene	100.000	99.750	0.250	0.999	0.997	0.998	3576	1.182	2.272	1.000	0.000028	99.750
DNA CNV	94.273	85.872	8.401	0.873	0.780	0.801	3315	5.651	3.142	0.969	0.000486	85.872
Parkinson’s disease	75.666	72.379	3.287	0.434	0.497	0.443	396	0.093	0.204	0.640	0.004378	72.379
Dermatology diseases	98.269	97.530	0.739	0.975	0.972	0.972	25	0.032	0.014	0.999	0.000752	97.530
BreastEW	92.007	91.569	0.438	0.930	0.895	0.904	22	0.064	0.021	0.979	0.004502	91.569
**RF classifier**												
RNA gene	100.000	99.502	0.498	0.997	0.994	0.996	3576	1.182	0.826	0.999	0.000410	99.502
DNA CNV	92.558	81.139	11.419	0.773	0.714	0.721	3315	5.651	1.584	0.944	0.000531	81.139
Parkinson’s disease	100.000	83.733	16.267	0.793	0.726	0.734	396	0.093	0.719	0.860	0.009057	83.733
Dermatology diseases	100.000	96.997	3.003	0.973	0.962	0.964	25	0.032	0.098	0.999	0.000567	96.997
BreastEW	99.982	96.140	3.842	0.961	0.959	0.958	22	0.064	0.118	0.986	0.002280	96.140
**Bagg classifier**												
RNA gene	99.940	99.126	0.814	0.996	0.990	0.992	3576	1.182	3.040	0.999	0.000260	99.126
DNA CNV	98.701	79.045	19.656	0.741	0.698	0.708	3315	5.651	1.095	0.911	0.000553	79.045
Parkinson’s disease	99.653	82.297	17.356	0.790	0.752	0.754	396	0.093	1.079	0.830	0.011270	82.297
Dermatology diseases	99.696	95.375	4.321	0.958	0.950	0.949	25	0.032	0.012	0.993	0.001466	95.375
BreastEW	99.636	95.253	4.383	0.957	0.946	0.948	22	0.064	0.029	0.987	0.001788	95.253

**Table 11:** The proposed methods compared with the mRMR method

Datasets	Train Data %	Test Data %	Over-fitting Diff. %	Pre	Rec	F1-score	NO.F	F-Time (sec)	C-Time (sec)	AUC	Var.	ACC %
**LR classifier**												
RNA gene	100.000	99.750	0.250	0.999	0.997	0.998	650	1200.011	0.251	1.000	0.000028	99.750
DNA CNV	91.819	79.699	12.120	0.746	0.688	0.689	505	2296.409	0.686	0.940	0.000529	79.699
Parkinson’s disease	74.617	73.011	1.606	0.500	0.515	0.479	145	61.005	0.017	0.659	0.002502	73.011
Dermatology diseases	95.508	95.075	0.433	0.950	0.908	0.919	15	3.996	0.002	0.995	0.000796	95.075
BreastEW	93.085	92.620	0.465	0.936	0.910	0.917	19	4.181	0.002	0.981	0.003358	92.620
**SVM classifier**												
RNA gene	100.000	99.748	0.252	0.999	0.997	0.998	650	1200.011	0.382	1.000	0.000028	99.748
DNA CNV	92.486	83.848	8.638	0.845	0.747	0.766	505	2296.409	3.609	0.961	0.000559	83.848
Parkinson’s disease	75.661	72.379	3.282	0.435	0.497	0.443	145	61.005	0.142	0.639	0.004378	72.379
Dermatology diseases	52.793	52.185	0.608	0.325	0.463	0.363	15	3.996	0.053	0.948	0.002302	52.185
BreastEW	89.049	88.938	0.111	0.915	0.860	0.870	19	4.181	0.044	0.945	0.005405	88.938
**RF classifier**												
RNA gene	100.000	99.627	0.373	0.998	0.996	0.997	650	1200.011	0.398	1.000	0.000036	99.627
DNA CNV	90.959	79.935	11.024	0.727	0.690	0.689	505	2296.409	0.534	0.942	0.001249	79.935
Parkinson’s disease	100.000	81.918	18.082	0.767	0.703	0.709	145	61.005	0.467	0.833	0.011138	81.918
Dermatology diseases	100.000	97.553	2.447	0.981	0.968	0.972	15	3.996	0.1000	0.999	0.000561	97.553
BreastEW	100.00	95.604	4.396	0.960	0.950	0.952	19	4.181	0.183	0.991	0.002693	95.604
**Bagg classifier**												
RNA gene	99.961	98.746	1.215	0.991	0.984	0.986	650	1200.011	1.680	0.999	0.000430	98.746
DNA CNV	97.817	77.468	20.349	0.731	0.682	0.687	505	2296.409	1.135	0.910	0.000937	77.468
Parkinson’s disease	99.498	79.369	20.129	0.725	0.712	0.705	145	61.005	0.561	0.799	0.010620	79.369
Dermatology diseases	99.545	94.017	5.528	0.951	0.936	0.937	15	3.996	0.009	0.982	0.002412	94.017
BreastEW	99.642	93.684	5.958	0.943	0.928	0.931	19	4.181	0.042	0.980	0.002369	93.684

The proposed methods were compared with many different filters methods as cited in the introduction section included in CfsSubsetEval, ReliefAttributeEval, OneRAttributeEval, ConsistencySubsetEval and PCA methods. Tables 12, 13, 14, 15 and 16 showed the results of these different filters methods. The ReliefAttributeEval method achieved the best results for RNA gene and BreastEW datasets, while ConsistencySubsetEval method gave the best results for DNA CNV dataset. In addition, CfsSubsetEval method gave the best results for Parkinson’s disease dataset, while the PCA method gave the best results for dermatology erythemato-squamous diseases dataset. Although filter methods improved the results, they did not give better results than the PFBS-RFS-RFE.

**Table 12:** The proposed methods compared with the CfsSubsetEval method

Datasets	Train Data %	Test Data %	Over-fitting Diff. %	Pre	Rec	F1-score	NO.F	F-Time (sec)	C-Time (sec)	AUC	Var.	ACC %
**J48 classifier**												
RNA gene	99.154	97.125	2.029	0.973	0.974	0.972	4083	3.860	1.030	0.987	0.000627	97.125
DNA CNV	64.034	63.682	0.352	0.539	0.537	0.533	41	2.950	0.009	0.815	0.000997	63.682
Parkinson’s disease	87.683	78.421	9.262	0.730	0.673	0.691	119	0.180	0.016	0.713	0.005994	78.421
Dermatology diseases	74.438	73.776	0.662	0.533	0.598	0.547	9	0.150	0.0002	0.884	0.002967	73.776
BreastEW	94.083	90.865	3.218	0.905	0.902	0.902	3	0.050	0.0002	0.950	0.000596	90.865
**Naïve base classifier**												
RNA gene	99.861	98.503	1.358	0.986	0.979	0.981	4083	3.860	0.048	0.987	0.000131	98.503
DNA CNV	64.864	64.196	0.668	0.601	0.620	0.598	41	2.950	0.001	0.884	0.001219	64.196
Parkinson’s disease	80.904	79.915	0.989	0.753	0.681	0.695	119	0.180	0.001	0.762	0.005410	79.915
Dermatology diseases	62.360	61.441	0.919	0.573	0.628	0.561	9	0.150	0.0007	0.935	0.005554	61.441
BreastEW	93.556	92.973	0.583	0.932	0.921	0.924	3	0.050	0.0008	0.979	0.000886	92.973
**K-nearest neighbors(KNN) classifier**												
RNA gene	99.792	99.627	0.165	0.998	0.996	0.997	4083	3.860	0.007	1.000	0.000036	99.627
DNA CNV	78.986	72.599	6.387	0.691	0.626	0.630	41	2.950	0.00009	0.862	0.000289	72.599
Parkinson’s disease	89.021	80.282	8.739	0.751	0.694	0.708	119	0.180	0.0008	0.743	0.002007	80.282
Dermatology diseases	85.247	79.767	5.48	0.823	0.783	0.780	9	0.150	0.0004	0.943	0.006681	79.767
BreastEW	87.503	81.172	6.331	0.815	0.793	0.797	3	0.050	0.0008	0.857	0.005639	81.172

**Table 13:** The proposed methods compared with the ReliefAttributeEval method

Datasets	Train Data %	Test Data %	Over-fitting Diff. %	Pre	Rec	F1-score	NO.F	F-Time (sec)	C-Time (sec)	AUC	Var.	ACC %
**J48 classifier**												
RNA gene	99.154	97.625	1.529	0.980	0.979	0.979	10,000	1.950	2.887	0.992	0.000432	97.625
DNA CNV	65.322	63.922	1.4	0.546	0.548	0.540	8000	1.550	0.994	0.816	0.001225	63.922
Parkinson’s disease	83.858	74.759	9.099	0.635	0.599	0.593	300	0.950	0.060	0.710	0.013702	74.759
Dermatology diseases	79.356	78.701	0.655	0.570	0.647	0.591	20	0.500	0.0007	0.924	0.000719	78.701
BreastEW	96.485	93.499	2.986	0.467	0.448	0.455	16	0.350	0.003	0.959	0.002994	93.499
**Naïve base classifier**												
RNA gene	99.855	96.881	2.974	0.962	0.955	0.955	10,000	1.950	0.115	0.973	0.000976	96.881
DNA CNV	65.678	64.679	0.999	0.629	0.668	0.620	8000	1.550	0.297	0.849	0.001359	64.679
Parkinson’s disease	81.550	79.338	2.212	0.742	0.729	0.722	300	0.950	0.003	0.775	0.014727	79.338
Dermatology diseases	87.403	85.570	1.833	0.808	0.852	0.806	20	0.500	0.0007	0.979	0.002454	85.570
BreastEW	94.681	94.415	0.266	0.481	0.444	0.460	16	0.350	0.0005	0.989	0.002167	94.415
**K-nearest neighbors(KNN) classifier**												
RNA gene	99.875	99.873	0.002	0.999	0.999	0.999	10,000	1.950	0.016	1.000	0.000031	99.873
DNA CNV	80.735	74.246	6.489	0.708	0.655	0.654	8000	1.550	0.013	0.874	0.000378	74.246
Parkinson’s disease	85.801	71.708	14.093	0.595	0.586	0.579	300	0.950	0.0007	0.608	0.007203	71.708
Dermatology diseases	92.289	86.059	6.23	0.872	0.848	0.840	20	0.500	0.001	0.961	0.004704	86.059
BreastEW	94.005	91.739	2.266	0.462	0.427	0.442	16	0.350	0.0002	0.964	0.000553	91.739

**Table 14:** The proposed methods compared with the OneRAttributeEval method

Datasets	Train Data %	Test Data %	Over-fitting Diff. %	Pre	Rec	F1-score	NO.F	F-Time (sec)	C-Time (sec)	AUC	Var.	ACC %
**J48 classifier**												
RNA gene	99.154	97.625	1.529	0.978	0.980	0.978	7000	2.021	1.779	0.991	0.000502	97.625
DNA CNV	65.367	64.059	1.308	0.543	0.557	0.542	5000	1.020	0.644	0.813	0.001261	64.059
Parkinson’s disease	85.627	78.744	6.883	0.741	0.670	0.673	200	0.150	0.038	0.764	0.012796	78.744
Dermatology diseases	79.448	77.080	2.368	0.563	0.633	0.578	15	0.120	0.0006	0.909	0.001517	77.080
BreastEW	96.641	92.095	4.546	0.919	0.912	0.914	17	0.105	0.002	0.962	0.001380	92.095
**Naïve base classifier**												
RNA gene	99.917	93.631	6.286	0.940	0.912	0.916	7000	2.021	0.076	0.949	0.000920	93.631
DNA CNV	67.368	66.528	0.840	0.617	0.625	0.610	5000	1.020	0.195	0.850	0.001035	66.528
Parkinson’s disease	74.262	73.784	0.478	0.400	0.434	0.415	200	0.150	0.001	0.715	0.009473	73.784
Dermatology diseases	86.004	83.589	2.415	0.812	0.791	0.758	15	0.120	0.0009	0.958	0.001873	83.589
BreastEW	93.451	93.196	0.250	0.469	0.460	0.462	17	0.105	0.001	0.986	0.003089	93.196
**K-nearest neighbors(KNN) classifier**												
RNA gene	99.723	99.627	0.096	0.998	0.996	0.997	7000	2.021	0.010	0.999	0.000036	99.627
DNA CNV	78.155	71.777	6.378	0.651	0.603	0.597	5000	1.020	0.009	0.842	0.000189	71.777
Parkinson’s disease	80.688	72.481	8.207	0.388	0.443	0.414	200	0.150	0.0001	0.623	0.002529	72.481
Dermatology diseases	87.431	84.700	2.731	0.787	0.787	0.780	15	0.120	0.002	0.963	0.003370	84.700
BreastEW	94.728	92.976	1.752	0.930	0.921	0.924	17	0.105	0.001	0.961	0.000953	92.976

**Table 15:** The proposed methods compared with the ConsistencySubsetEval method

Datasets	Train Data %	Test Data %	Over-fitting Diff. %	Pre	Rec	F1-score	NO.F	F-Time (sec)	C-Time (sec)	AUC	Var.	ACC %
**J48 classifier**												
RNA gene	93.106	91.136	1.97	0.880	0.869	0.869	3	1.850	0.001	0.963	0.000641	91.136
DNA CNV	64.842	63.921	0.921	0.550	0.549	0.543	42	1.600	0.012	0.816	0.001172	63.921
Parkinson’s disease	86.346	80.279	6.067	0.765	0.688	0.707	11	1.100	0.003	0.738	0.002407	80.279
Dermatology diseases	87.918	87.740	0.178	0.751	0.755	0.742	12	0.102	0.0006	0.946	0.002407	87.740
BreastEW	96.993	94.380	2.613	0.950	0.933	0.939	8	0.090	0.0008	0.971	0.000873	94.380
**Naïve base classifier**												
RNA gene	97.545	97.380	0.165	0.972	0.970	0.970	3	1.850	0.001	0.994	0.000188	97.380
DNA CNV	73.015	71.606	1.409	0.682	0.698	0.680	42	1.600	0.006	0.923	0.001276	71.606
Parkinson’s disease	76.940	75.516	1.424	0.666	0.599	0.605	11	1.100	0.0005	0.729	0.003051	75.516
Dermatology diseases	90.265	89.839	0.426	0.878	0.901	0.867	12	0.102	0.0007	0.995	0.002740	89.839
BreastEW	94.630	94.201	0.429	0.943	0.934	0.937	8	0.090	0.002	0.988	0.000686	94.201
**K-nearest neighbors(KNN) classifier**												
RNA gene	97.517	97.131	0.386	0.963	0.964	0.962	3	1.850	0.001	0.993	0.000311	97.131
DNA CNV	82.735	77.059	5.676	0.750	0.680	0.685	42	1.600	0.0001	0.888	0.000465	77.059
Parkinson’s disease	79.100	69.698	9.402	0.557	0.524	0.517	11	1.100	0.002	0.583	0.002962	69.698
Dermatology diseases	97.632	95.916	1.716	0.962	0.943	0.947	12	0.102	0.0008	0.994	0.001339	95.916
BreastEW	95.704	93.496	2.208	0.937	0.925	0.929	8	0.090	0.0009	0.965	0.000621	93.496

**Table 16:** The proposed methods compared with the PCA method

Datasets	Train Data %	Test Data %	Over-fitting Diff. %	Pre	Rec	F1-score	NO.F	F-Time (sec)	C-Time (sec)	AUC	Var.	ACC %
**J48 classifier**												
RNA gene	96.823	94.884	1.939	0.943	0.954	0.942	700	1.027	0.174	0.985	0.000740	94.884
DNA CNV	62.334	60.665	1.669	0.526	0.516	0.505	2800	49.795	1.549	0.827	0.001724	60.665
Parkinson’s disease	83.745	73.816	9.929	0.625	0.607	0.602	250	0.079	0.028	0.645	0.002418	73.816
Dermatology diseases	81.148	80.878	0.270	0.576	0.663	0.604	18	0.016	0.003	0.925	0.000142	80.878
BreastEW	95.723	93.493	2.230	0.942	0.925	0.929	20	0.016	0.002	0.959	0.000832	93.493
**Naïve base classifier**												
RNA gene	87.072	79.403	7.669	0.794	0.806	0.794	700	1.027	0.005	0.954	0.002116	79.403
DNA CNV	29.336	27.641	1.695	0.255	0.352	0.233	2800	49.795	0.077	0.680	0.000319	27.641
Parkinson’s disease	74.471	73.821	0.65	0.604	0.558	0.545	250	0.079	0.009	0.698	0.002826	73.821
Dermatology diseases	98.361	96.179	2.182	0.961	0.952	0.953	18	0.016	0.0001	0.997	0.001025	96.179
BreastEW	90.041	89.803	0.238	0.896	0.886	0.889	20	0.016	3.057	0.962	0.001707	89.803
**K-nearest neighbors(KNN) classifier**												
RNA gene	99.750	99.740	0.010	0.999	0.997	0.998	700	1.027	0.002	0.999	0.000059	99.740
DNA CNV	81.200	74.348	6.852	0.663	0.639	0.634	2800	49.795	0.010	0.867	0.000273	74.348
Parkinson’s disease	81.158	72.612	8.546	0.612	0.571	0.575	250	0.079	0.001	0.627	0.002308	72.612
Dermatology diseases	92.380	87.162	5.218	0.862	0.860	0.844	18	0.050	0.00002	0.969	0.001984	87.162
BreastEW	94.728	92.976	1.752	0.930	0.921	0.924	20	0.030	0.0003	0.961	0.000953	92.976

Table 17 showed the comparison between the proposed methods, MIFS, CBF and FCBF methods as cited in the introduction section. The CBF gave the best results for RNA gene dataset, while FCBF method gave the best results for DNA CNV, Parkinson’s disease and BreastEW datasets. In addition, MIFS gave the best results for dermatology erythemato-squamous diseases dataset. These methods did not give the best results when compared with the PFBS-RFS-RFE.

**Table 17:** The proposed methods compared with the MIFS, CBF and FCBF methods

Datasets	Train Data %	Test Data %	Over-fitting Diff. %	Pre	Rec	F1-score	NO.F	F-Time (sec)	C-Time (sec)	AUC	Var.	ACC %
**Mutual Information**												
**KNN classifier**												
RNA gene	99.736	99.627	0.109	0.998	0.996	0.997	10,000	258.902	0.008	1.000	0.000036	99.627
DNA CNV	82.686	76.097	6.589	0.745	0.663	0.667	9000	180.314	0.011	0.854	0.000368	76.097
Parkinson’s disease	80.879	72.479	8.400	0.610	0.568	0.572	300	2.121	0.0001	0.624	0.002344	72.479
Dermatology diseases	97.966	97.267	0.699	0.975	0.969	0.969	25	0.351	0.002	0.963	0.000839	97.267
BreastEW	94.435	92.628	1.807	0.927	0.917	0.920	20	0.083	0.00002	0.958	0.001419	92.628
**Correlation Based Feature**												
**KNN classifier**												
RNA gene	99.867	99.748	0.119	0.999	0.997	0.998	900	2.600	0.003	1.000	0.000092	99.748
DNA CNV	52.831	49.073	3.758	0.447	0.402	0.369	750	1.850	0.003	0.669	0.000490	49.073
Parkinson’s disease	81.158	72.612	8.546	0.612	0.571	0.575	320	0.255	0.002	0.627	0.002308	72.612
Dermatology diseases	94.171	90.953	3.218	0.871	0.855	0.846	20	0.202	0.002	0.947	0.002243	90.953
BreastEW	94.747	92.976	1.771	0.931	0.920	0.924	17	0.105	0.002	0.961	0.000953	92.976
**Fast Correlation Based Feature**												
**KNN classifier**												
RNA gene	99.742	99.625	0.117	0.998	0.996	0.997	400	1.750	0.001	1.000	0.000131	99.625
DNA CNV	81.390	76.236	5.154	0.721	0.671	0.676	13	0.800	0.007	0.905	0.001131	76.236
Parkinson’s disease	82.657	73.270	9.387	73.271	0.585	0.587	16	1.500	0.002	0.675	0.001767	73.270
Dermatology diseases	97.936	97.005	0.931	0.970	0.967	0.966	14	0.101	0.002	0.961	0.001217	97.005
BreastEW	95.333	95.078	0.255	0.953	0.945	0.947	7	0.006	0.002	0.953	0.000261	95.078

Table 18 showed the proposed methods compared with the Chi-square method as cited in the introduction section using SVM and RF classifiers. The SVM classifiers gave the best results for RNA gene and DNA CNV datasets, while RF classifier gave the best results for, Parkinson’s disease, BreastEW and dermatology erythemato-squamous diseases datasets. This method did not give the best results when compared with the PFBS-RFS-RFE.

**Table 18:** The proposed methods compared with the Chi-square method

Datasets	Train Data %	Test Data %	Over-fitting Diff. %	Pre	Rec	F1-score	NO.F	F-Time (sec)	C-Time (sec)	AUC	Var.	ACC %
**SVM classifier**												
RNA gene	100.000	99.625	0.375	0.997	0.995	0.996	7555	0.0801	2.379	1.000	0.000036	99.625
DNA CNV	79.862	70.130	9.732	0.592	0.586	0.584	5555	0.528	3.050	0.901	0.000369	70.130
Parkinson’s disease	75.661	72.228	3.433	0.471	0.497	0.448	398	0.016	0.210	0.628	0.000814	72.228
Dermatology diseases	71.220	70.488	0.732	0.556	0.653	0.565	24	0.094	0.093	0.653	0.001305	70.488
BreastEW	91.994	91.563	0.431	0.929	0.895	0.906	21	0.016	0.016	0.976	0.001014	91.563
**RF classifier**												
RNA gene	100.000	99.502	0.498	0.997	0.995	0.996	7555	0.0801	1.009	1.000	0.000041	99.502
DNA CNV	86.934	68.552	18.382	0.585	0.572	0.570	5555	0.528	2.817	0.891	0.000240	68.552
Parkinson’s disease	100.000	81.087	18.913	0.755	0.701	0.704	398	0.016	0.471	0.836	0.008783	81.087
Dermatology diseases	100.000	98.355	1.645	0.984	0.981	0.982	24	0.094	0.229	0.998	0.000363	98.355
BreastEW	100.000	96.832	3.168	0.973	0.962	0.965	21	0.016	0.104	0.990	0.001265	96.832

Table 19 showed the proposed methods compared with the IGF, Chi-square and Bat algorithm as cited in the introduction section. The Bat algorithm gave the best results for RNA gene, DNA CNV and BreastEW datasets, while Chi-square method gave the best results for Parkinson’s disease dataset. In addition, the IGF method gave the best results for dermatology erythemato-squamous diseases dataset. These methods did not give the best results when compared with the PFBS-RFS-RFE.

**Table 19:** The proposed methods compared with the IGF, Chi-square and Bat algorithm methods

Datasets	Train Data %	Test Data %	Over-fitting Diff. %	Pre	Rec	F1-score	NO.F	F-Time (sec)	C-Time (sec)	AUC	Var.	ACC %
**Information gain**												
**KNN classifier**												
RNA gene	99.723	99.627	0.096	0.998	0.996	0.997	3576	1.182	0.005	1.000	0.000036	99.627
DNA CNV	81.310	74.448	6.862	0.671	0.640	0.636	3315	5.651	0.014	0.850	0.001361	74.448
Parkinson’s disease	81.026	72.479	8.547	0.777	0.885	0.827	396	0.093	0.0008	0.452	0.002384	72.479
Dermatology diseases	97.996	97.267	0.729	0.974	0.970	0.969	25	0.032	0.0002	0.964	0.000496	97.267
BreastEW	94.728	92.976	1.752	0.924	0.888	0.904	22	0.064	0.002	0.969	0.000953	92.976
**Naïve base classifier**												
RNA gene	100.000	96.380	3.620	0.9685	0.946	0.952	3576	1.182	0.029	0.970	0.000710	96.380
DNA CNV	66.994	65.637	1.357	0.647	0.657	0.626	3315	5.651	0.435	0.832	0.001336	65.637
Parkinson’s disease	74.618	74.070	0.548	0.803	0.867	0.833	396	0.093	0.004	0.721	0.006235	74.070
Dermatology diseases	86.947	85.781	1.166	0.828	0.856	0.802	25	0.032	0.0007	0.984	0.001143	85.781
BreastEW	94.259	93.853	0.406	0.946	0.887	0.914	22	0.064	0.002	0.988	0.000766	93.853
**Decision tree classifier**												
RNA gene	99.154	97.250	1.904	0.975	0.977	0.975	3576	1.182	0.836	0.989	0.000444	97.250
DNA CNV	65.626	64.574	1.052	0.553	0.559	0.551	3315	5.651	2.067	0.820	0.000900	64.574
Parkinson’s disease	86.449	75.528	10.921	0.810	0.878	0.841	396	0.093	0.077	0.707	0.005645	75.528
Dermatology diseases	88.494	85.015	3.479	0.725	0.745	0.721	25	0.032	0.0008	0.934	0.003209	85.015
BreastEW	96.466	94.029	2.437	0.924	0.920	0.918	22	0.064	0.003	0.967	0.001310	94.029
**Chi-square**												
**KNN classifier**												
RNA gene	99.847	99.750	0.097	0.999	0.997	0.998	7555	0.0801	0.010	1.000	0.000028	99.750
DNA CNV	70.283	59.635	10.648	0.526	0.498	0.492	5555	0.528	0.005	0.753	0.002142	59.635
Parkinson’s disease	81.158	72.612	8.546	0.778	0.886	0.828	398	0.016	0.001	0.452	0.002308	72.612
Dermatology diseases	92.622	88.498	4.124	0.889	0.875	0.865	24	0.094	0.0009	0.967	0.002641	88.498
BreastEW	94.728	92.976	1.752	0.924	0.888	0.904	21	0.016	0.002	0.969	0.000953	92.976
**Naïve base classifier**												
RNA gene	100.000	75.787	24.213	0.746	0.683	0.680	7555	0.0801	0.184	0.809	0.004833	75.787
DNA CNV	49.600	48.765	0.835	0.512	0.506	0.468	5555	0.528	0.552	0.734	0.000893	48.765
Parkinson’s disease	74.691	74.207	0.484	0.797	0.879	0.835	398	0.016	0.006	0.708	0.008073	74.207
Dermatology diseases	89.445	87.164	2.281	0.816	0.860	0.817	24	0.094	0.0004	0.975	0.001954	87.164
BreastEW	94.220	93.678	0.542	0.946	0.882	0.912	21	0.016	0.0007	0.988	0.000967	93.678
**Decision tree classifier**												
RNA gene	99.154	97.250	1.904	0.974	0.976	0.974	7555	0.0801	5.221	0.990	0.000410	97.250
DNA CNV	58.451	55.863	2.588	0.464	0.458	0.452	5555	0.528	2.155	0.776	0.000253	55.863
Parkinson’s disease	83.127	76.721	6.406	0.823	0.878	0.849	398	0.016	0.117	0.729	0.005338	76.721
Dermatology diseases	88.494	85.015	3.479	0.725	0.745	0.721	24	0.094	0.0008	0.934	0.003209	85.015
BreastEW	96.466	93.327	3.139	0.915	0.911	0.909	21	0.016	0.010	0.966	0.002513	93.327
**Bat algorithm**												
**KNN classifier**												
RNA gene	99.861	99.752	0.109	0.999	0.997	0.998	6483	1350	0.012	1.000	0.000027	99.752
DNA CNV	81.786	75.309	6.477	0.685	0.644	0.639	5301	1280	0.008	0.864	0.000235	75.309
Parkinson’s disease	80.277	69.326	10.951	0.757	0.869	0.809	35	0.305	9.422	0.507	0.003475	69.326
Dermatology diseases	98.027	97.260	0.767	0.971	0.970	0.969	19	0.255	0.002	0.974	0.001678	97.260
BreastEW	94.728	92.976	1.752	0.924	0.888	0.904	14	0.200	0.001	0.969	0.000953	92.976
**Naïve base classifier**												
RNA gene	99.882	83.777	16.105	0.858	0.787	0.792	6483	1350	0.084	0.875	0.002498	83.777
DNA CNV	67.463	66.290	1.173	0.654	0.663	0.632	5301	1280	0.186	0.873	0.002012	66.290
Parkinson’s disease	75.617	74.744	0.873	0.792	0.899	0.841	35	0.305	0.0008	0.706	0.005885	74.744
Dermatology diseases	86.109	85.540	0.569	0.801	0.850	0.799	19	0.255	0.001	0.979	0.001915	85.540
BreastEW	95.477	95.099	0.378	0.962	0.906	0.931	14	0.200	0.0005	0.990	0.001183	95.099
**Decision tree classifier**												
RNA gene	99.320	98.750	0.570	0.988	0.990	0.988	6483	1350	1.782	0.994	0.000139	98.750
DNA CNV	65.592	64.608	0.984	0.553	0.560	0.551	5301	1280	0.578	0.822	0.000894	64.608
Parkinson’s disease	81.820	74.609	7.211	0.783	0.915	0.843	35	0.305	0.010	0.699	0.003413	74.609
Dermatology diseases	89.011	87.177	1.834	0.747	0.761	0.742	19	0.255	0.002	0.942	0.002909	87.177
BreastEW	96.466	94.029	2.437	0.924	0.920	0.918	14	0.200	0.003	0.968	0.001310	94.029

Table 20 showed the comparison between the PFBS-RFS-RFE and other filter ones methods. The results showed that the PFBS-RFS-RFE gave the best results when compared with other filter ones methods.

**Table 20:** The comparison between the PFBS-RFS-RFE and other filter ones methods

Algorithm	ACC%	NO.F	Pre	Rec	F1-score	AUC	Var.
MIFS	99.875	10,000	0.999	0.998	0.988	1.000	0.000016
IGF	99.875	3576	0.999	0.999	0.998	1.000	0.000016
mRMR	99.750	650	0.999	0.997	0.998	1.000	0.000028
CfsSubsetEval	99.627	4083	0.998	0.996	0.997	1.000	0.000036
ReliefAttributeEval	99.873	10,000	0.999	0.999	0.999	1.000	0.000031
OneRAttributeEval	99.627	7000	0.998	0.996	0.997	0.999	0.000036
ConsistencySubsetEval	97.380	3	0.972	0.970	0.970	0.994	0.000188
PCA	99.740	700	0.999	0.997	0.998	0.999	0.000059
MIFS, CBF and FCBF	99.748	900	0.999	0.997	0.998	1.000	0.000092
Chi-square	99.625	7555	0.997	0.995	0.996	1.000	0.000036
IGF, Chi-square and Bat algorithm	99.752	6483	0.999	0.997	0.998	1.000	0.000027
Proposed method (PFBS-RFS-RFE)	100.000	10.000	1.000	1.000	1.000	1.000	0.0

The proposed methods were compared with some hybrid-recursive feature elimination methods as cited in the introduction section. Table 21 showed the results of the hybrid-recursive feature elimination methods for all datasets using RFE and LR. The results proved that this hybrid method gave the best results for RNA Gene, dermatology erythemato-squamous diseases and BreastEW datasets. This hybrid method did not give the best results when compared with the PFBS-RFS-RFE.

**Table 21:** The proposed methods compared with the hybrid of MIFS and RFE

Datasets	Train Data %	Test Data %	Over-fitting Diff. %	Pre	Rec	F1-score	NO.F	F-Time (sec)	C-Time (sec)	AUC	Var.	ACC %
**RF classifier**												
RNA gene	100.000	99.501	0.499	0.794	0.715	0.723	5000	10,227.579	1.199	1.000	0.000041	99.501
DNA CNV	92.908	85.034	7.874	0.770	0.716	0.717	4500	88,434.627	3.411	0.946	0.000698	85.034
Parkinson’s disease	100.000	83.861	16.139	0.809	0.737	0.759	150	74.445	0.411	0.876	0.002867	83.861
Dermatology diseases	99.727	94.819	4.908	0.941	0.930	0.930	12	1.113	0.079	0.996	0.001528	94.819
BreastEW	100.000	95.965	4.035	0.961	0.953	0.956	10	1.592	0.133	0.988	0.000756	95.965

Another hybrid method is applied to show the comparison between the proposed method and hybrid method using GA and RFE. Table 22 showed the results of the hybrid method using GA and RFE. The results proved that this hybrid method gave the best results for RNA gene and BreastEW datasets. This hybrid method did not give the best result when compared with the PFBS-RFS-RFE.

**Table 22:** The proposed methods compared with the hybrid of GA and RFE

Datasets	Train Data %	Test Data %	Over-fitting Diff. %	Pre	Rec	F1-score	NO.F	F-Time (sec)	C-Time (sec)	AUC	Var.	ACC %
**SVM classifier**												
RNA gene	99.791	99.750	0.221	0.999	0.997	0.998	3123	15,746.043	0.727	1.000	0.000028	99.750
DNA CNV	93.271	84.980	8.291	0.860	0.770	0.790	2940	62,405.810	35.118	0.965	0.000620	84.980
Parkinson’s disease	75.529	74.996	0.533	0.530	0.523	0.474	149.000	55.114	0.071	0.768	0.000652	74.996
Dermatology diseases	84.800	84.722	0.078	0.854	0.835	0.830	5.000	0.651	0.016	0.960	0.000052	84.722
BreastEW	91.799	91.394	0.405	0.463	0.420	0.439	5.000	0.656	0.016	0.977	0.000776	91.394

In addition, the proposed method was compared with another hybrid method using ridge regression and RFE. Table 23 showed the results of the hybrid method using ridge regression and RFE. The results proved that this hybrid method gave the best results for RNA gene, dermatology erythemato-squamous diseases and BreastEW datasets. This hybrid method did not give the best result when compared with the PFBS-RFS-RFE.

**Table 23:** The proposed methods compared with the hybrid of Ridge regression and RFE

Datasets	Train Data %	Test Data %	Over-fitting Diff. %	Pre	Rec	F1-score	NO.F	F-Time (sec)	C-Time (sec)	AUC	Var.	ACC %
**SVM classifier**												
RNA gene	100.000	99.627	0.373	0.998	0.830	0.831	10,265	10,160.720	2.962	1.000	0.000036	99.627
DNA CNV	93.446	80.761	12.685	0.772	0.707	0.710	8190	37,302.17	3.216	0.944	0.000527	80.761
Parkinson’s disease	100.000	82.930	17.070	0.810	0.714	0.738	376.000	5.482	1.195	0.855	0.003410	82.930
Dermatology diseases	99.727	94.805	4.922	0.950	0.947	0.944	13.000	0.016	0.080	0.994	0.001556	94.805
BreastEW	100.000	93.675	6.325	0.941	0.926	0.932	15.000	0.0159	0.101	0.984	0.000969	93.675

Table 24 showed the comparison between the PFBS-RFS-RFE and other RFE hybrid methods. The results showed that the PFBS-RFS-RFE gave the best results when compared with other RFE hybrid methods.

**Table 24:** The comparison between the PFBS-RFS-RFE and other RFE hybrid methods

Algorithm	ACC%	NO.F	Pre	Rec	F1-score	AUC	Var.
MIFS and RFE	99.501	4500	0.794	0.715	0.723	1.000	0.000041
GA and RFE	99.750	3123	0.999	0.997	0.998	1.000	0.000028
Ridge regression and RFE	99.627	10,265	0.998	0.830	0.831	1.000	0.000036
Proposed method (PFBS-RFS-RFE)	100.000	10.000	1.000	1.000	1.000	1.000	0.0

After the number of runs, the selected features are intersected to know the genes (features) associated with cancers which considered the most developing human cancers. Table 25 presented the features after the intersection, which played an important role in knowing the most genes and features developing human cancers.

**Table 25:** The selected features after intersection [38–58]

**Datasets**	**No.** **Intersection** **Features**	**Feature indices** **or feature names**	**Feature or gene Description**	**Reference** **in cancer**
**RNA gene**	1	G110	–	–
**DNA** **CNV**	12	PPP1R8	Through alternative splicing, three this gene encodes different isoforms [38].	[39]
		SCARNA1	Small Cajal body-specific RNA 1 [38].	[40]
		RPA2	Protein A (RPA) complex is encoded by this gene [38].	[41]
		SMPDL3B	Sphingomyelin phosphodiesterase acid like 3B [38].	[42]
		XKR8	Promotes phosphatidylserine exposure apoptotic cell surface, possibly by mediating phospholipid scrambling [43].	[44]
		PHACTR4	A member of the phosphatase and actin regulator (PHACTR) family are encoded by this gene [38].	[45]
		RCC1	Regulator of chromosome condensation 1 [38].	[46]
		SNHG3	Small nucleolar RNA host gene 3 [32].	[47]
		SNORD99	Small nucleolar RNA, C/D box 99 [38].	[48]
		SNORA16A	Small nucleolar RNA, H/ACA box 16A [38].	[49]
		RAB42	Member RAS oncogene family [38].	–
		TFA12	This gene Control of transcription by RNA polymerase II [38].	[50]
**Parkinson’s** **disease**	7	IMF_SNR_TKEO	–	–
		IMF_NSR_TKEO	–	–
		mean_MFCC_1st_coef	–	–
		mean_4th_delta_delta	–	–
		mean_5th_delta_delta	–	–
		mean_6th_delta_delta	–	–
		mean_7th_delta_delta	–	–
**BreastEW**	1	Radius	Can be defined as the mean of distances from center to points on the perimeter [51].	[51]
**Datasets**	**No.** **Intersection** **Features**	**Feature indices** **or feature names**	**Feature or gene Description**	**Reference** **in derma-** **tology**
**Dermatology erythemato-squamous diseases**	5	Borders	The border of the lesion which important for diagnosing and for other features [52, 53].	[53]
		Parakeratosis	Nucleated keratinocytes are existed in the stratum corneum due to accelerated keratinocytic turnover [54].	[54]
		Spongiosis	Intraepidermal eosinophils is existed in spongiotic zones [55].	[55, 56]
		Itching	Itching is a bad feeling that causes itching continuously, which affects the human psyche [57].	[56]
		Age	The age at disease onset [58].	[58]

For DNA CNV dataset, the PHACTR4 was associated with prostate, breast and colon cancer [59], while RPA2 was associated with breast cancer [41]. We can notice that the proposed method achieved the best results and reached the most effective genes that develop human cancer. For dermatology erythemato-squamous diseases dataset, the age, itching and spongiosis features were associated with psoriasis dis- ease [56, 58].

## Discussion

The proposed PFBS-RFS-RFE was applied to classify different human cancer using big, medium and small datasets and other medical dataset. It used five different datasets. PFBS-RFS-RFE was proposed to enhance drawbacks included in over-fitting, time-consuming, high dimension, variance and classification accuracy. The PFBS was applied in different position to obtain different results. It was applied using three positions outer, inner and outer/inner. After applying PFBS, the RFS algorithm for feature selection was applied to select the most relevant features and reduce time consumption in RFE algorithm. RFE algorithm was used to obtain the final relevant subset of features with higher classification accuracy results.

The OFBS-RFS-RFE method achieved the best results using all datasets. The RF classifier achieved the best classification accuracy with 100% using dermatology erythemato-squamous diseases dataset with 0.0 variance results. The features and time were reduced to become 16.000 and 0.500, respectively. Furthermore, LR classifier achieved the best classification accuracy result with 99.981% using RNA gene dataset, while the SVM classifier gave the best variance result with 0.0000002. The number of features and time were reduced to become 142.500 and 0.192 s, respectively. From DNA CNV dataset the difference between training and testing was reduced using LR and Bagg classifiers, and the accuracy results were increased with 91.020 and 92.762%, respectively using the same classifiers. In addition, the OFBS-RFS-RFE reduced the variance between features to become 0.00028 and 0.00023, respectively, using the previous classifiers. The number of features and time were reduced to become 675 and 2.147 s, respectively.

From Parkinson’s disease dataset the classification accuracy and variance are enhanced to become 95.000% and 0.00062, respectively using RF classifier. The features were reduced to 113.85 features which well enough for classification step with 1.134 s as a computational time. From BreastEw dataset the best computational time was after applying LR and SVM in contrast with the other optimizer. The RF gave the best variance and accuracy to become 0.000302 and 98%, respectively. The features and time were reduced to become 0.070 and 0.070 s, respectively.

The IFBS-RFS-RFE not achieves the best results in all datasets. The SVM classifier achieved the best classification accuracy and variance results from the RNA gene dataset with 99.988% and 0.0000002, respectively. The features and time were minimized to 125.25 features and 0.153 s, respectively. For other datasets it did not give good results.

The O/IFBS-RFS-RFE achieved the best results for dermatology erythemato-squamous diseases dataset. RF and Bagg classifiers gave the best results with 10 features. The classification accuracy, variance and time were improved to become 100%, 0.0 and 0.500, respectively. In addition, The O/IFBS-RFS-RFE achieved the best results in high dimension datasets using RNA gene. The LR classifier increased the accuracy and variance results to 99.994% and 0.0000004, respectively. From DNA CNV dataset, the Bagg classifier gave the best accuracy and variance results to become 92.834% and 0.00027, respectively. At the same time, the outer/inner position did not provide good results for other datasets.

For future work, our proposed method will apply the incremental feature selection (IFS) for different datasets using PFBS. The IFS will select the most relevant subset features to minimize the time when using all features and overcome the feature selection drawback.

## Conclusions

In our study, new hybrid methods are proposed to enhance cancers classification performance using different size of datasets. The PFBS using EDF equation is enhanced the RFS and RFE performance. Many bootstrap positions are applied to improve the problem of over-fitting and to fix the feature selection problems. Furthermore, our proposed methods achieved high results using different size of datasets. It is compared with previous work and it gave high results.

## Method

### Dataset description

We used five healthcare datasets in the experiments. The DNA CNV dataset is used in [7, 8, 12] and downloaded from the cBioPortal for Cancer Genomics [59–61] to classify different types of human cancers. The other four datasets are downloaded from the UCI machine learning repository [62] and used in [9, 23]. A brief description of each adopted dataset is presented in Table 26.

**Table 26:** Datasets Description

Category Type	DS No.	Datasets	#Features	#Samples	#Class
Small < 100	D1	BreastEW	30	569	2
	D2	Dermatology erythemato-squamous diseases	34	366	6
Medium 100 < D2 < 1000	D3	Parkinson’s disease	753	756	2
Large 1000 < D < 21,000	D4	DNA CNV	16,381	2916	6
	D5	RNA gene	20,531	801	5

### The proposed hybrid feature selection methods

**Fig. 4:**
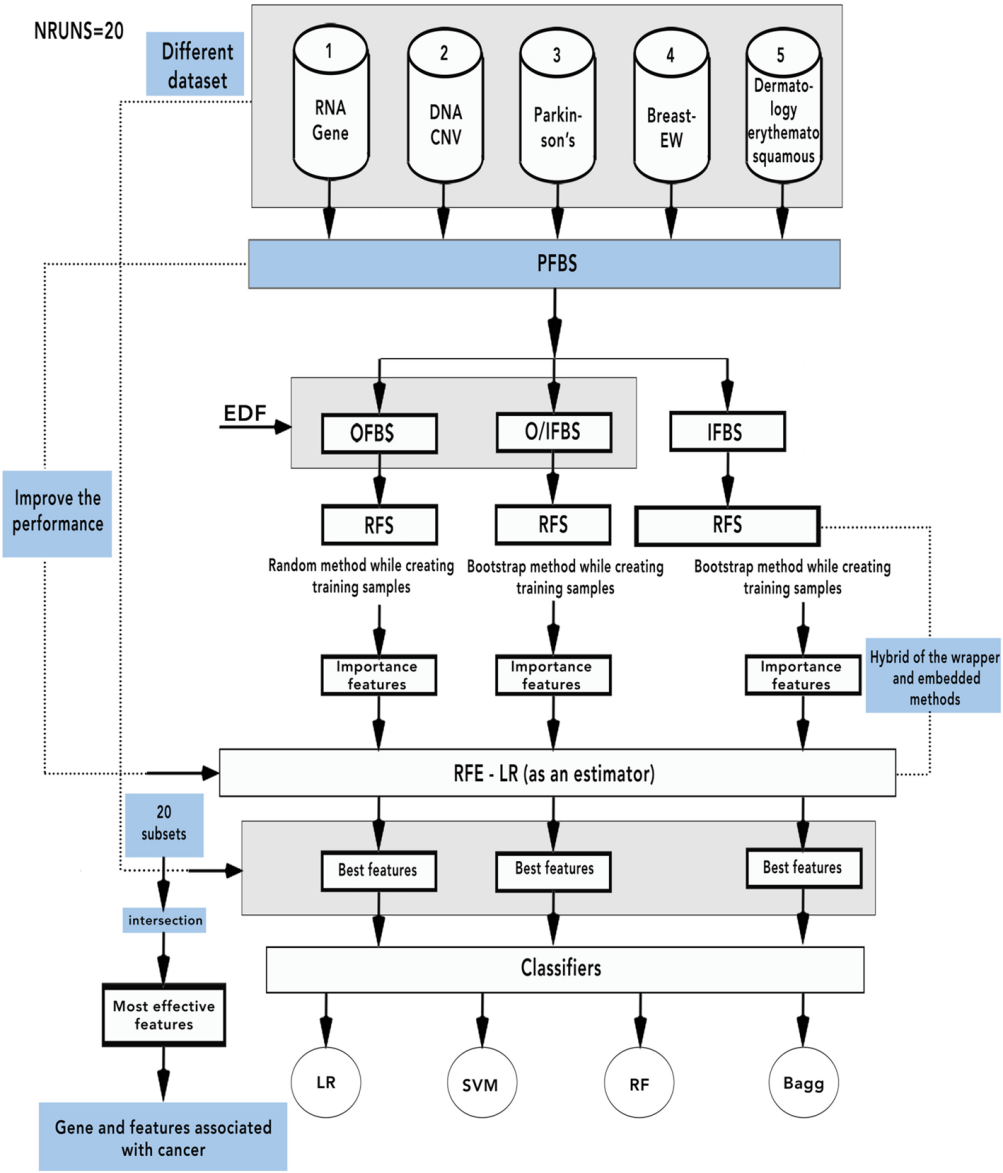
Hybrid proposed methods for feature selection

The main motivation of the proposed methods is to select the most important and relevant features from all original features. This step is considered vital and plays a significant role in obtaining good classification results. Non- influencing features waste time and lead to many complex problems included in poor classification accuracy, over-fitting, and feature size. The wrapper method for feature selection selects the features based on machine learning to find optimal features, but it takes more time to obtain these features and has chances of over-fitting problems. On the other hand, the advantage of embedded methods for feature selection is that the selected features are embedded in machine learning or during the model building process. It is applied to reduce the over-fitting problem, reducing the variance between features. Based on the advantages of the two previous methods, we proposed hybrid methods for feature selection to obtain the most relevant subset feature. The proposed methods are shown in Fig. 4. Resampling method with different positions is applied to minimize the over-fitting problem and maximize the classification accuracy. After the resampling step, the most important features are selected using RFS algorithm. The hybrid between resampling and RF algorithms are applied to solve many problems such as (1) time consuming when using RFE algorithm, (2) over-fitting problem, (3) the most relevant features, and (4) classification accuracy. The wrapper method is applied to select the most important features, therefore; reduce the datasets dimensional and maximizing the classification accuracy. The RFE using LR classification as an estimator is integrated with the previous features to achieve the desired goals.

### First bootstrap step as a resampling method

A lot of high-dimensional datasets suffer from over-fitting problems and low classification accuracy. We apply the FBS step as a resampling method to avoid these problems. The bootstrap samples are drawn with replacement as the same size of the original data. Given the original datasets X = X_1_, X_2_, X_3_, ........, X_O_ With O observations with a distribution function called empirical distribution function (EDF). The bootstrap sample is denoted as X^*^ = X^*^_1_, X^*^_2_, X^*^_3_, ......., X^*^_O_. The (EDF) is denoted as follows [63]: -

5 $${\hat{F}}_O(t)=\sum_{I=1}^OI\left({X}_i\le t\right)/O$$

Where I(·) denotes the indicator function, the bootstrap resampling method is applied in many positions to achieve the desired task. The first position of bootstrap is before selecting the essential features called OFBS, but we need to apply different positions to obtain the best results. In this position the EDF is applied as a resampling method before selecting features. The IFBS is applied during selecting the feature selection. On the other hand, the O/IFBS is applied before and during selecting features. All bootstrap positions are applied to overcome the over-fitting and classification accuracy. After these positions, the classification accuracy and over-fitting problems are improved. Therefore, the proposed positions selected the most relevant features.

### Feature selection using random Forest (RFS)

A random forest algorithm is applied for feature selection to improve the performance of the classifiers, reduce the over-fitting problem and time consuming due to the disadvantage of RFE algorithm. It is considered the embedded feature selection that interacts directly with classifiers and reduces the time complexity found in the wrapper method. The RFS algorithm can identify the importance of the feature. The training samples are created using bootstrap when applying IFBS method but using all datasets to create samples when applying OFBS to improve the over-fitting and classification accuracy. The trees are constructed with a specific size. Select M trees from the dataset to build the decision trees. Decision trees are constructed from the M trees and they are repeated B times. Construct the smallest subset of features F at each node and separate the best features for F by Gini importance scores. It is sorted the features according to their scores from smallest to largest. The features below the threshold will be eliminated.

### Recursive feature elimination (RFE)

Selecting the most significant features is the main goal in the classification step. In this direction, we applied RFE algorithm to select the most important features therefore; reach to the chromosome which considered the most developing human cancers. RFE is an instance of backward feature elimination. The classifier estimator is trained on the initial set of features and these features are sorted according to their weights. The features with the smallest weights are removed because these features are not important during the classification process. The previous steps are repeated until the most relevant features are reached. RFE is applied with LR as an estimator. The classification accuracy is improved after applying the proposed method. The step size is proposed in the RFE method called recursive feature elimination with cross-validation (RFECV) to achieve the best results. The features are sorted according to their importance at each step, and the smallest ranked feature is deleted. The proposed methods are presented in Tables 27, 28 and 29 as follows:

**Table 27:**
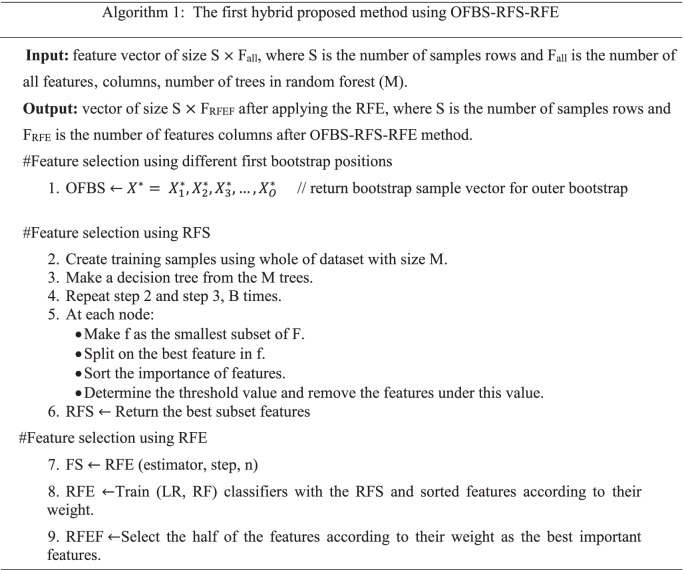
Algorithm 1 of the first hybrid proposed method using OFBS-RFS-RFE

**Table 28:**
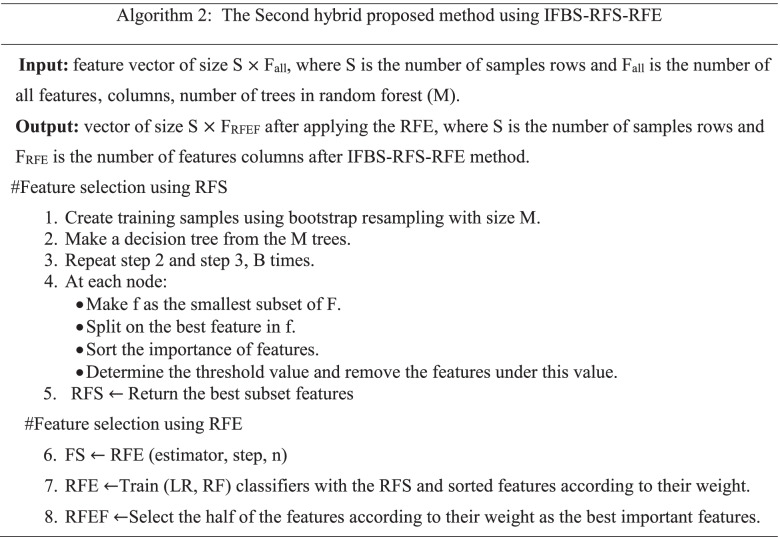
Algorithm 2 of the second hybrid proposed method using IFBS-RFS-RFE

**Table 29:**
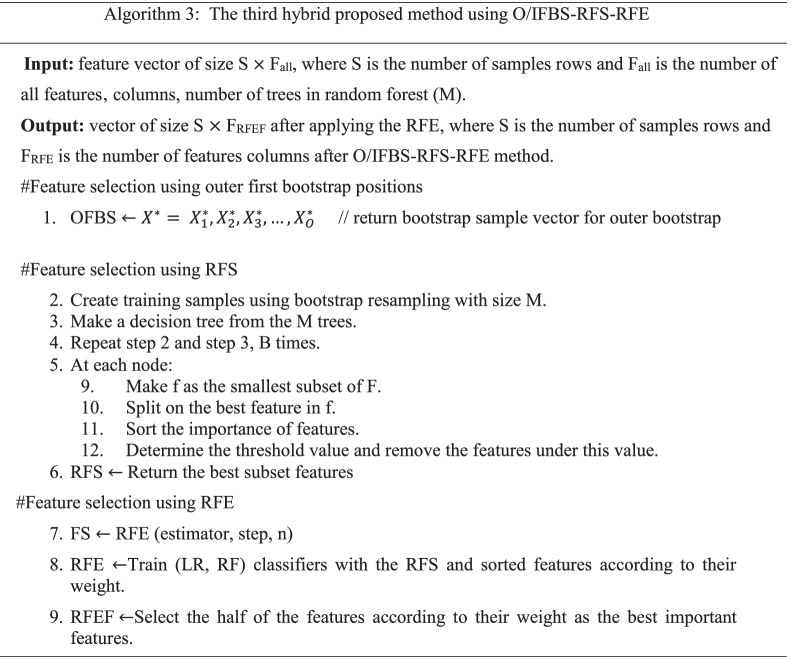
Algorithm 3 of the third hybrid proposed method using O/IFBS-RFS-RFE

## Data Availability

All datasets and details are available at request from the corresponding author and as a supplement to this article.

## Abbreviations

RFE: Recursive feature elimination
RFS: Random forest for selection
PFBS: Positions first bootstrap step
PFBS-RFS-RFE: Positions first bootstrap step random forest selection recursive feature elimination
OFBS: Outer first bootstrap step
IFBS: Inner first bootstrap step
O/IFBS: Outer/Inner first bootstrap step
MIFS: Mutual information based feature
IGF: Information gain based feature selection
CNV: Copy Number Variation
LR: Logistic regression
SVM: Support vector machine
PCA: Principal component analysis
CBF: Correlation based feature
FCBF: Fast correlation based feature selection
KNN: K-nearest neighbors
SSA: Salp swarm algorithm
CSSA: Constant salp swarm algorithm
PSO: Particle swarm optimization
GA: Genetic algorithm
LLSVM: Linear support vector machine
GBM-RFE: Gradient boosting machines RFE
BPSO: Binary particle swarm optimization
mRMR: Minimum redundancy maximum relevance
IFS: Incremental feature selection
SFLA: Shuffled frog leaping algorithm
EDF: Distribution function called empirical distribution function
RFECV: Recursive feature elimination with cross-validation
ROC: Receiver operating characteristic
PPV: Positive predictive value
TP: True positive
TN: True negative
FN: False-negative
FP: False-positive
